# Knowledge, perceptions and practices of health students and professionals regarding leishmaniasis in Portugal: a cross-sectional study

**DOI:** 10.1186/s13071-023-05982-z

**Published:** 2023-10-25

**Authors:** Rafael Rocha, Cláudia Conceição, Luzia Gonçalves, Carla Maia

**Affiliations:** 1 Instituto de Higiene e Medicina Tropical (IHMT), Universidade Nova de Lisboa (UNL), Lisbon, Portugal; 2 Global Health and Tropical Medicine (GHTM), Associate Laboratory in Translation and Innovation Towards Global Health (LA-REAL), Institute of Hygiene and Tropical Medicine (IHMT), NOVA University Lisbon (UNL), Lisbon, Portugal; 3 Centro de Estatística e Aplicações da Universidade de Lisboa (UL), Lisbon, Portugal; 4 Z-Stat4life, Lisbon, Portugal

**Keywords:** Leishmaniasis, Awareness, Knowledge, Perceptions, Practices, Medicine, Veterinary, Environmental health, Portugal, One Health

## Abstract

**Background:**

Control of leishmaniasis in the Mediterranean Basin relies on the active contributions from researchers in the fields of animal, human and environmental health. The application of knowledge, perceptions and practices (KPP) questionnaires to health students and professionals in Europe could be fundamental to identify and explore gaps in KPP, highlighting the diversity of conceptions related to this disease between students and professionals active in (One) Health. The objective of this study was to characterize and compare the current knowledge, perceptions and practices regarding leishmaniasis among subgroups of students and health professionals in Portugal through the application of an online questionnaire.

**Methods:**

A cross-sectional study targeted the population of health students and professionals in Portugal, including students in medicine, veterinary medicine and environmental health, physicians, veterinarians and environmental health technicians. Potential participants were approached by email via universities and professional societies and organizations and provided with the link to access the online questionnaire. Answers to the self-administered sociodemographic and KPP questionnaire were collected between July and December 2022. Individual KPP scores were calculated by summing grades defined for each question. Logistic regression models were used to search for potential associations, and the results were expressed at estimated crude and adjusted odds ratios with 95% confidence intervals.

**Results:**

In total, 486 participants were included in this study: 254 students and 232 professionals. Overall, 75% of the participants reported having heard of both human and animal leishmaniasis, and > 80% reported hearing about the disease during their course work (although this was significantly lower among those in the field of environmental health). Around 90% of participants identified the pathogenic agent as a parasite, and an arthropod bite was identified as the main route of transmission by > 95%. Animal leishmaniasis was considered to be diagnosed in Portugal by 87% of participants and human leishmaniasis by only 69%. The main barriers pointed out by professionals to the control of leishmaniasis were: lack of knowledge in the general population, failures in the early diagnosis and treatment of diseased animals, absence/inefficacy of vector control programs and lack of knowledge in human health professionals. Median knowledge and perception scores were higher among professionals in the animal health field and higher in professionals than in students. Median practice scores were not significantly different between groups and subgroups. The multivariate analysis revealed that a longer period of study (for students) and having seen cases of leishmaniasis (for physicians) were associated with above-mentioned median knowledge score.

**Conclusions:**

Most health students and professionals are knowledgeable about the cause and transmission route of leishmaniasis. However, recognition of the disease as autochthonous in humans is less common, highlighting the importance of promoting an approach to this infection through a One-Health lens. A national structured plan to control leishmaniasis could overcome some of the barriers pointed out by professionals, namely by implementing systematic phlebotomine surveillance and integrated reporting of animal and human cases of disease.

**Graphical abstract:**

**Supplementary Information:**

The online version contains supplementary material available at 10.1186/s13071-023-05982-z.

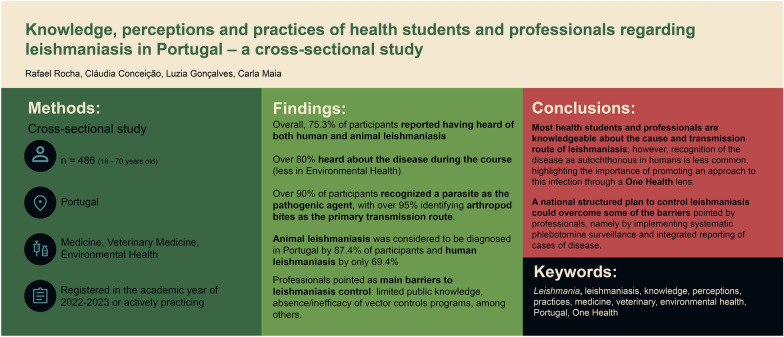

## Background

Leishmaniases are a group of diseases caused by protozoan parasites of the *Leishmania* genus that are transmitted through the bite of phlebotomine sand flies. In southern Europe, including Portugal, *Leishmania infantum* is the only endemic human pathogenic species and is maintained in zoonotic cycles where dogs are the most important domestic reservoirs [1]. Human infection by parasites of the *Leishmania* genus is asymptomatic in most cases [2]. However, some individuals progress to clinically recognizable disease, which can be grouped in the following syndromic forms: visceral leishmaniasis (VL), cutaneous leishmaniasis (CL) and mucosal leishmaniasis (ML/MCL). In Portugal, symptomatic infection by the endemic species *L. infantum* most often results in VL [3], and the reporting of VL cases to central public health authorities is mandatory. Between 2014 and 2018, 6–14 cases were reported each year nationwide [4], although these numbers likely reflect a significant underreporting at the hospital level, as the numbers were higher in previous periods [5]. CL is considered to be rare in Portugal and is not listed as a mandatory declaration disease, so available data of cases are dispersed and mostly derived from the few case reports published in national and international literature [6]. Regarding canine leishmaniasis (CanL), there is also no national, integrated and standardized reporting and surveillance system in place, and only cases suspected by municipal veterinarians during rabies control campaigns are reported to the General Directorate for Food and Veterinary; consequently, the number of cases diagnosed annually at a global and regional level is unknown [3]. However, seroprevalence studies performed in dogs, at both national and regional levels, have provided some insight into the distribution of infection, helping to define regions where exposure to *Leishmania* parasites is expected to be more frequent/intense, namely in the districts of Beja, Portalegre and Castelo Branco [7, 8].

Despite the wide distribution of leishmaniasis and the health impacts on the population in endemic areas, knowledge, perceptions and practices (KPP) regarding this disease are not homogeneous between countries and between regions of the same country, or even different sectors of the population, including health professionals, health sciences students and animal owners. Studies to assess the knowledge, perceptions, attitudes and practices regarding VL have been conducted mostly in South Asia [9–11[, South America [12, 13] and East Africa [14], with the target study populations consisting predominantly of resident communities in highly endemic areas. In the Mediterranean region, where VL is also endemic, the few studies dedicated to analyzing knowledge, attitudes and practices (KAP) of the resident general population were mostly directed to animal owners, including three studies performed in Portugal [15–17]. With respect to studies directed to the population of health professionals, there is an abundant body of literature on the knowledge and practices of veterinary doctors in Mediterranean countries, including Portugal, generally with a focus on the epidemiology and clinical approach to CanL [18–21]. However, no studies carried out in Mediterranean countries have included medical doctors and environmental health technicians (EHTs) in the target population. On a worldwide perspective, few studies have addressed these groups, although it is generally recognized that they play an important role in controlling leishmaniasis, under a One Health lens. In a number of studies, physicians were enrolled in completing structured questionnaires, either self-administered (online or paper) or by interview, in endemic areas such as South Asia [22, 23], North Africa [24, 25], Middle East [26, 27] and South America [28, 29]. Both primary healthcare physicians and specialists (such as dermatologists) were included in these studies. Some published studies included educational interventions, showing a significant increase in knowledge following the intervention [26, 28]. These studies showed that knowledge on the cause, transmission route, clinical presentation, diagnosis, treatment and perception of risk were very diverse among regions. Even though the designation EHT is not employed homogeneously across countries, professionals involved in environmental health were involved in at least one study in Brazil [30] in which correct answers were compared between different professional groups; The highest average score was achieved by veterinarians, followed by physicians and EHTs, although few participants were recruited in each group. Some observational and interventional studies targeted high-school students in countries such as Iran and Ethiopia [26, 31, 32]. University students in health sciences, including medicine, veterinary medicine and environmental health, have only rarely been included in KAP/KPP studies and never in the context of a Mediterranean country, as shown in a recent review article on CL [33]; however, an understanding of the current knowledge and perceptions of future professionals could be an essential step towards raising awareness and improving practices in the health community. One study involving medical students in Latin America participating in an online questionnaire [34] showed that most students were aware that leishmaniasis was caused by a parasite transmitted by sand flies and recognized ways of preventing the disease, but they were less knowledgeable regarding the clinical presentation of disease and treatment.

In Portugal, incomplete reporting and insufficient characterization of symptomatic leishmaniasis cases (human and canine) may result in gaps in KPP regarding leishmaniasis in health professionals. These gaps may be perpetuated due to neglect of leishmaniasis in the training of students in these areas. The application of KPP questionnaires to students and professionals could be fundamental to identifying and exploring these gaps, highlighting the diversity of conceptions related to this disease between students and professionals in (One) Health. Therefore, the aim of this project was to characterize and compare current KPP regarding leishmaniasis among subgroups of students and health professionals, in Portugal, through the application of an online questionnaire, in a sample of each of these groups.

## Methods

### Study population and sample size calculation

This cross-sectional, observational study was carried out from July to December 2022, in Portugal, which is located in southwest Europe, bordering Spain and the Atlantic Ocean. The study consisted of the self-administration of a structured, anonymous, online questionnaire aimed at collecting sociodemographic data and information on KPP related to leishmaniasis. The populations targeted were students of medicine, veterinary medicine and environmental health in Portuguese public and private higher education institutions as well as health professionals (medical doctors, veterinary doctors and EHTs) working in public and private institutions in Portugal.

The most recent statistics of the Portuguese Order of Physicians show that 59,545 professionals were registered in 2021 [35]. Most of these were women (56.7%), and the districts where most professionals worked were Lisbon (29.0%), Porto (22.1%) and Coimbra (9.7%) [35]. The age groups with the highest proportion of registered doctors < 31 years (17.4%) and > 65 years (24.0%). The medical specialties that could potentially be involved in a more detailed approach to leishmaniasis represented a significant fraction of the registered specialists: Internal Medicine (8.7%, *n* = 3165), Pediatrics (6.3%, *n* = 2297), Public Health (1.6%, *n* = 582), Dermatology (1.2%, *n* = 427) and Infectious Diseases (0.6%, *n* = 227) [36]. It should be noted that at least 1.1% of registered doctors completed their medical training in countries or regions where cases of leishmaniasis are rare or absent, such as Portuguese-speaking African countries, non-Mediterranean European countries, North America and Oceania [37]. Based on data from the National Institute of Statistics, 12,449 medical students were registered in 2021, with a predominance of women (69.7%), of whom 18.4% were registered for the first time in that year [38]. These students were registered in programs offered by eight faculties (7 public and 1 private), located in five Portuguese cities (Braga, Coimbra, Covilhã, Lisboa and Porto), according to data available in the Directorate General for Higher Education (DGES) [39].

Statistics from the Portuguese Order of Veterinary Doctors show that 6788 active members were registered in 2022, most of whom were women (65.2%). In terms of distribution by regions, most actively working members were in the districts of Lisbon (26.3%, *n* = 1783), Porto (15.6%, *n* = 1057) and Setúbal (8.2%, *n* = 556) [40]. Based on data from the DGES, 2959 veterinary medicine students were registered in the 2019–2020 academic year, most of whom were (77.0%). Veterinary medicine courses are currently offered in eight faculties (4 public and 4 private) located in six Portuguese cities (Almada, Coimbra, Évora, Lisboa, Porto and Vila Real) [39].

Lastly, the global number of EHT professionals in Portugal could not be ascertained via official available sources. However, based on data from the DGES, there were 387 environmental health students (EHSs) registered in the 2019–2020 academic year, with a predominance of women (70.3%) [39]. This bachelor’s course is offered in three public institutes, located in the cities of Coimbra, Lisbon and Porto.

The sample size and geographic distribution of each student/professional group were not determined a priori and depended on the rates of participation from each institution. However, a standardized protocol was used to approach potential participants in each group in terms of number, frequency, media and content of contacts, as explained in the following text. To ensure a nationwide coverage of sampling, collaboration in this study was proposed to all of the faculties and institutes where the target courses were available, as detailed above, and to the professional orders, societies and associations of the three targeted professional categories.

### Eligibility criteria

Individuals included in this study cumulatively fulfilled the following criteria:

- Being registered (in Portugal) as a student in the academic year of 2022–2023 in one of three degree programs, namely Integrated Master’s in Medicine, Integrated Master’s in Veterinary Medicine or Bachelor’s in Environmental Health, or having completed training in medicine, veterinary medicine or environmental health and actively practicing (in Portugal) in these professional fields in 2022.
- Age between 18 and 70 years, inclusive.
- Access to an electronic device for filling in the online questionnaire.
- Consenting to the informed consent form to participate in the study.

### Data and sample collection

All of the higher education institutions in Portugal offering the Integrated Master’s in Medicine, Integrated Master’s in Veterinary Medicine and Bachelor’s in Environmental Health were contacted with requests for collaboration in disseminating the questionnaire to the students enrolled in these courses. Collaboration was achieved in four of the eight medical faculties, five of th eight veterinary faculties and two of the three environmental health institutes. The link to access the questionnaire was sent to all students enrolled in these courses via their institutional emails; this first email was followed by two subsequent emails at 3 and 6 weeks after the first. Additionally, the Portuguese Order of Physicians (OM), the Portuguese Order of Veterinary Doctors (OMV) and the Portuguese Association of Environmental Health (APSAi) were contacted and requested to disseminate the questionnaire among their registered professionals or members. For the APSAi, we used the same approach as that for the faculties. For the OMV, however, the link to the questionnaire was posted one the APSAi website and, due to low visibility and adherence, the questionnaire was also disseminated through posting in specific Facebook® groups, following a similar timeline (3 posts separated by 3 weeks). Collaboration of the OM was not possible, so professional medical societies were contacted to request their collaboration (specialties of Infectious Diseases, Pediatrics, Anatomopathology, Clinical Pathology, Dermatology, Internal Medicine and Public Health). Only the first three specialties collaborated, and the questionnaire was sent via email to the associates following a similar timeline; this was complemented by posting in specific Facebook® medical groups.

The questionnaire was constructed specifically for this study, although some questions were adapted from previous KPP research on leishmaniasis. The questions were designed to address all of the relevant topics regarding knowledge on leshmaniasis (epidemiology, presentation, diagnosis and management aspects) and professional and personal practices, while avoiding redundancy. Questions considered to be appropriate for the purpose of the project based on consensus of all authors were included in the final version of the questionnaire. To improve ease/speed of filling in the questionnaire, most questions were designed as multiple choice; possible answers were selected by the authors to include all “correct” knowledge based on current scientific evidence and all the expected most common practices; additional plausible options were added to allow greater discrimination. Likert scales were used as answers to perception questions.

The questionnaire was pre-tested on a convenience sample of health students and professionals and readapted to achieve conformity. The first page of the form consisted of an information text and request for consent. Progression required consent, selection of the professional/student category and confirmation of current activity. Participants went on to fill in a self-administered online questionnaire about sociodemographic and professional/academic aspects and KPP regarding leishmaniasis. This questionnaire was built upon RedCap® (Research Electronic Data Capture—a secure web application for building and managing online surveys and databases), and different versions were available for different groups, although many questions overlapped, and some were directed specifically to one or a few groups (Additional file 1: Figure S1). The latter was the case for professional activity-related questions and for questions targeting biological details of vectors. Additionally, some questions were only visible to participants who selected specific answers to previous questions, in a drop-off fashion. For most questions, an answer was not mandatory for progressing to the next question in the questionnaire. The total number of accesses to the questionnaire was registered, but only submitted forms were saved and used for analysis.

Categorical variables extracted from the questionnaire were analyzed mostly using the original categories provided as answer options, but regrouping was performed in some cases. Districts of work/study in mainland Portugal were grouped into five regions (Norte, Centro, Lisboa e Vale do Tejo [LVT], Alentejo and Algarve) according to the areas of activity of the five Regional Coordination and Development Commissions (Comissões de Coordenação e Desenvolvimento Regional [CCDR]). For questions answered in an ordinal scale with *k* options, answers were rated from 0 to *k* (with the *k* value attributed to the highest frequency, importance or agreement).

### Statistical analysis

Absolute and relative frequencies, hypothesis testing and logistic regressions were performed using IBM® SPSS® Statistics Version 29.0 (SPSS IBM Corp, Armonk, NY, USA). Bar and pie charts were built using Microsoft® Excel® (Microsoft Corp., Redmond, WA, USA).

Answers to each KPP question were scored according to the criteria presented in Additional file 2: Table S1. A total score for each individual was calculated for knowledge (K score), for perceptions (Per score) and for practices (Pra score), by totaling the scores for all the questions in each category. The range of possible values for each score was 0–8 for K, 0–11 for Per and 0–3.5 for Pra.

Descriptive statistics were expressed as absolute frequencies and percentages for categorical variables and as means with standard deviations or medians with interquartile ranges for continuous variables (e.g. age, and K, Per and Pra scores). Comparisons between groups were performed using the Pearson Chi-square test (*χ*^2^) for categorical variables (or Fisher’s exact test in case of failure of the assumptions of the *χ*^2^test). For continuous variables, after checking the assumptions of normality and homogeneity of the variances, instead of the t-test and analysis of variance (ANOVA), we used the Mann–Whitney U-test or the Kruskal–Wallis test for comparing ≥ 2 independent groups, respectively. For variables rated in an ordinal scale, the median value was calculated and presented in the tables, and statistical significance between groups was assessed comparing distributions using the Mann–Whitney U-test or the Kruskal–Wallis test (≥ 2 independent groups, respectively). A value of *P* < 0.05 was considered statistically significant.

Recoding into binary variables was performed. Participants were divided in two groups for K, Per and Pra scores: those with scores above the global median score value and those with scores equal or below this value (K = 6.5, Per = 9, Pra = 1.5). Multivariate analyses were conducted to identify sociodemographic and occupational factors associated with higher K, Per or Pra scores. These analyses were performed through multiple binary logistic regression models, analyzing variables with statistical meaning in the univariate analysis (*P* < 0.05) and some biologically relevant or potentially confounding variables. The reference categories used for each independent variable are specified in each multivariate analysis results table. For those variables that remained significant, crude odds ratios (ORs) were updated to adjusted odds ratios (aORs) with 95% confidence intervals (CIs). The Hosmer–Lemeshow test was used to assess goodness of fit in each multiple logistic regression model [41].

## Results

### Sociodemographic and occupational characteristics

In total, 486 consented to participate in the study, of whom 254 were students and 232 were professionals. The sociodemographic characteristics of these participants are summarized in Table 1. Median age was 21 years old for students and 38 years for professionals; differences in age were not statistically significant among subgroups of students nor among subgroups of health professionals. Female gender was predominant in all subgroups (> 70%). The distribution of participants by region was significantly different between students and professionals and among subgroups, but Lisboa e Vale do Tejo (LVT) and Norte were the most represented (except for EHSs).

**Table 1:** Sociodemographic characteristics of the participants, for students and professionals

Sociodemographic characteristics	Students					Professionals				
	Global	Medicine	Veterinary science	EH	*P-*value	Global	Physicians	Veterinarians	EH technicians	*P*-value
*n*	254	140	98	16		232	116	57	59	
Median age, years [IQR]	21 [19–24]	21 [19–23]	21 [20–24]	20.5 [20–21]	0.146 (*H* = 3.8,* df* = 2)	38 [30–46]	36 [30–46]	36 [30–46]	42.5 [32.25–45.75]	0.350 (*H* = 2.1,* df* = 2)
*Gender, % (n)*										
Male	16.7 (42/252)	20.1 (28/139)	12.4 (12/97)	12.5 (2/16)	0.259 (*χ*^2^ = 2.7,* df* = 2)	23.3 (54/232)	28.4 (33/116)	17.5 (10/57)	18.6 (11/59)	0.174 (*χ*^2^ = 3.5,* df* = 2)
Female	83.3 (210/252)	79.9 (111/139)	87.6 (85/97)	87.5 (14/16)		76.7 (178/232)	71.6 (83/116)	82.5 (47/57)	81.4 (48/59)	
*Region of work/study, % (n)*										
Norte	26.2 (66/252)	20.1 (28/139)	37.1 (36/97)	12.5 (2/16)	< 0.001* (FET = 70.5)	32.5 (75/231)	36.5 (42/115)	21.1 (12/57)	35.6 (21/59)	0.011* (FET = 21.0)
Centro	10.7 (27/252)	11.5 (16/139)	0 (0/97)	68.8 (11/16)		18.2 (42/231)	23.5 (27/115)	8.8 (5/57)	16.9 (10/59)	
LVT	55.6 (140/252)	68.3 (95/139)	43.3 (42/97)	18.7 (3/16)		38.5 (89/231)	33.0 (38/115)	54.4 (31/57)	33.9 (20/59)	
Alentejo	7.5 (19/252)	NA	19.6 (19/97)	NA		3.0 (7/231)	1.7 (2/115)	7.0 (4/57)	1.7 (1/59)	
Algarve	NA	NA	NA	NA		5.6 (13/231)	3.5 (4/115)	8.8 (5/57)	6.8 (4/59)	
Açores/Madeira	NA	NA	NA	NA		2.2 (5/231)	1.7 (2/115)	0 (0/57)	5.1 (3/59)	

*EH* Environmental Health,* FET* Fisher’s exact test,* IQR* interquartile range,* LVT* Lisboa e Vale do Tejo, *n* number,* NA* not applicable

*Statistically significant according to Chi-square test, FET or Kruskal-Wallis H-test, as shown

**Fig. 1:**
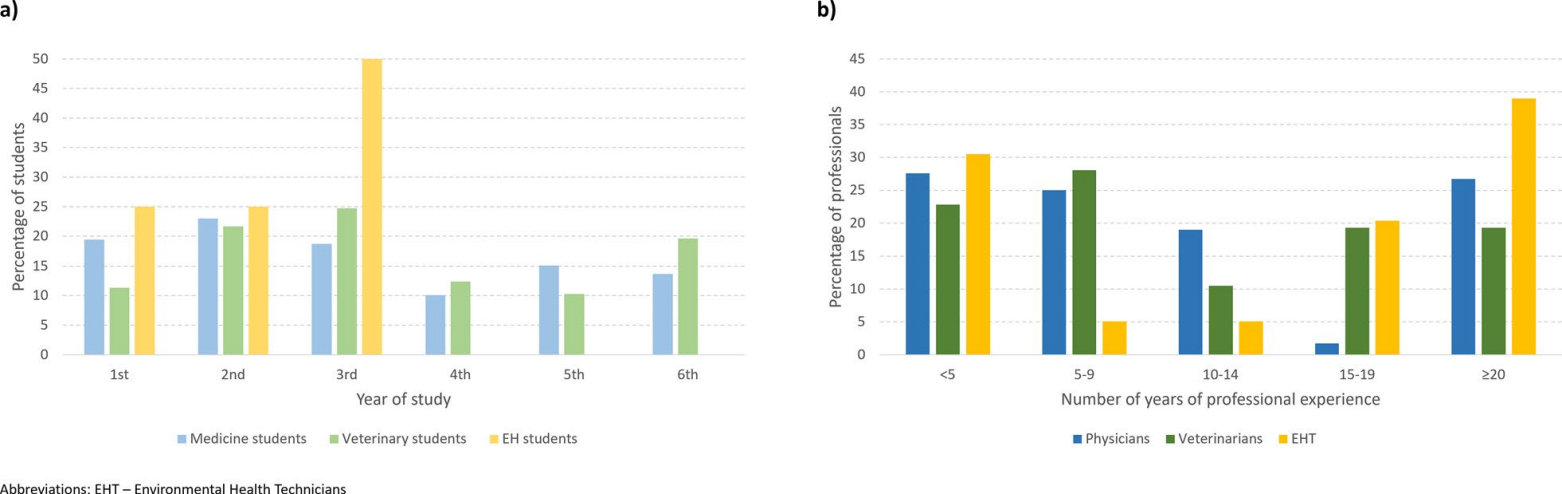
Distribution of (**a**) medical and veterinary students by year of study (**a**) and of professionals by number of years of professional experience (**b**)

Distribution of students by year of study is shown in Fig. 1a (for medicine and veterinary students: Chi-square test, *χ*^2^ = 5.9,* df* = 5, *P* = 0.324) and distribution of professionals by number of years of experience is shown in Fig. 1b (Kruskal–Wallis test,* H* = 3.5,* df* = 2, *P* = 0.170). Distribution of students by university and faculty is shown in Additional file 3: Figure S2. The type of work most performed by physicians and veterinarians was consultations (63.8% and 84.2%, respectively), followed by infirmary visits (62.1% and 42.1%, respectively). Most physicians were specialists (64.7%, *n* = 75) or specialty residents (31.9%, *n* = 37) and were practicing in the following specialties: Pediatrics (38.8%, *n* = 45), Infectious Diseases (20.7%, *n* = 24), Anatomopathology (8.6%, *n* = 10), Public Health (6.9%, *n* = 8), Internal Medicine and Family Medicine (5.2% each, *n* = 6), Clinical Pathology (3.4%, *n* = 4) and Dermatology (0.9%, *n* = 1). Types of institutions where most of the professionals worked were: Public Health Units (88.1%, *n* = 52) for EHTs; public hospitals (79.3%, *n* = 92), for physicians; veterinary clinics (78.9%, *n* = 45) for veterinarians. Most veterinarians reported working with companion animals (94.7%, *n* = 54), but also with livestock (12.3%, *n* = 7), exotic animals (10.5%, *n* = 6) and horses (7.0%, *n* = 4).

### Knowledge results

**Fig. 2:**
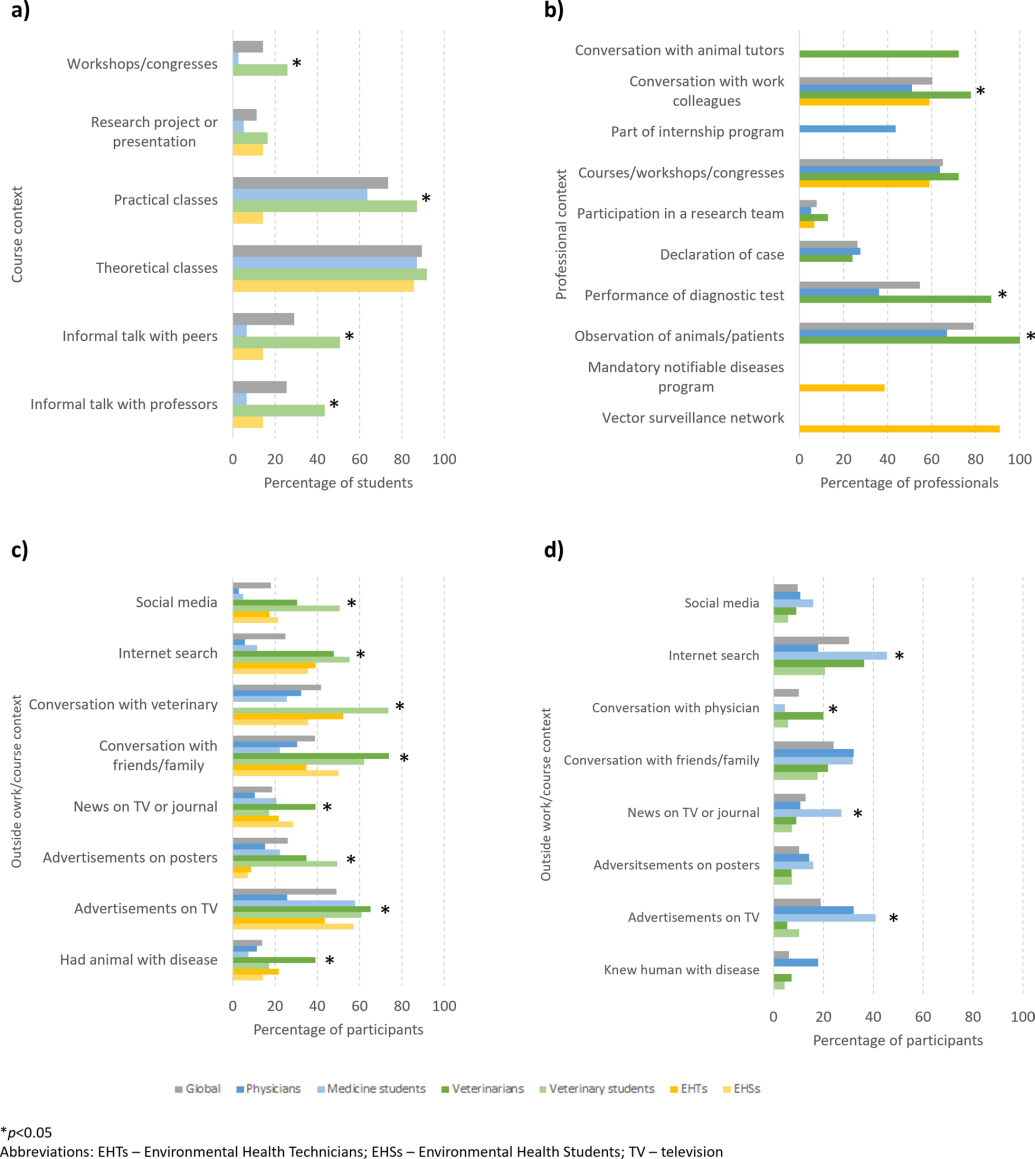
Percentage of participants, globally and by student/professional group, reporting having heard of leishmaniasis in different contexts: **a** during the course, **b** during professional activities, **c** outside the course/work (animal leishmaniasis), **d** outside the course/work (human leishmaniasis). Asterisk indicates a significant difference at *P < 0.05

Answers to individual knowledge questions are summarized in Table 2. Overall, 75.3% of participants reported having heard of both human and animal leishmaniasis, although this percentage was significantly higher in professionals (Fisher’s exact test, 49.8, *P* < 0.001). Over 80% of participants reported hearing about the disease during their courses; the number was similar between students and professionals globally, but was significantly lower among people in the field of EH (Chi-square test, *χ*^2^ = 14.2,* df* = 2, *P* = 0.001). The course year most reported by students for hearing of leishmaniasis was the second, both for veterinary students (71.1%) and for medical students (60.8%). Hearing about the disease during professional activity was more commonly reported by veterinarians (94.7%), followed by physicians (82.8%) and EHTs (77.2%). Hearing about animal leishmaniasis in professionals during their career in human was less frequently reported than hearing about human leishmaniasis careers in animal health (42.9% vs 77.7%, *P* < 0.001). Professionals reported hearing about leishmaniasis relatively less often outside their course/work (Chi-square test, *χ*^2^ = 63.2,* df* = 1, *P* < 0.001). Figure 2 presents the percentage of participants reporting hearing of leishmaniasis in different contexts at work, during their course work and outside of their work/course work. The most reported contexts for hearing of leishmaniasis at work were the observation of animals/patients, conversation with work colleagues, performance of diagnostic tests and courses/workshops/congresses; the first three categories were significantly more selected by veterinarians than by physicians. In course work, theoretical classes were the context most often selected, followed by practical classes; compared to medical and EH students, veterinary students reported hearing of leishmaniasis significantly more often in practical classes, workshops/congresses and informal talks with peers or professors. Outside of the work/course context, the two most reported contexts for hearing of leishmaniasis were advertisements on TV and conversation with a veterinary, for animal leishmaniasis; internet searches and conversation with friends/family, for human leishmaniasis.

**Table 2:** Answers to knowledge questions, globally and by student/professional group

Questions	All	Students					Professionals					*p*-value global
		Global	Medicine	Veterinary science	EH	*P*-value	Global	Physicians	Veterinarians	EHTs	*P*-value	
*Heard of leishmaniasis?*												
Animals only	17.7 (86/486)	28.3 (72/254)	26.4 (37/140)	29.6 (29/98)	37.5 (6/16)	0.094 (FET = 10.8)	6.0 (14/232)	2.6 (3/116)	3.5 (2/57)	11.9 (7/59)	0.004* (FET = 19.4)	< 0.001* (FET = 49.8)
Humans only	4.3 (21/486)	3.9 (10/254)	6.4 (9/140)	0 (0/98)	6.2 (1/16)		4.7 (11/232)	9.5 (11/116)	0 (0/57)	0 (0/59)		
Humans and animals	75.3 (366/486)	63.4 (161/254)	60.0 (84/140)	70.4 (69/98)	50.0 (8/16)		88.4 (205/232)	87.9 (102/116)	96.5 (55/57)	81.4 (48/59)		
No/DK/CR	2.7 (13/486)	4.3 (11/254)	7.1 (10/140)	0 (0/98)	6.2 (1/16)		0.9 (2/232)	0 (0/116)	0 (0/57)	6.8 (4/59)		
*Heard of leishmaniasis during course work?*												
Yes	82.4 (361/438)	82.0 (169/206)	82.8 (77/93)	86.7 (85/98)	46.7 (7/15)	0.001* (*χ*^2^ = 14.2,* df* = 2)	82.8 (192/232)	85.3 (99/116)	100 (57/57)	63.2 (36/57)	< 0.001* (FET = 26.1)	0.843 (*χ*^2^ = 0.04, * df* = 1)
*Heard of leishmaniasis outside of course work/work?*												
Yes	50.2 (220/438)	70.4 (145/206)	47.3 (44/93)	88.8 (87/98)	93.3 (14/15)	< 0.001* (FET = 43.4)	32.3 (75/232)	25.0 (29/116)	40.4 (23/57)	40.4 (23/57)	0.046* (*χ*^2^= 6.2,* df* = 2)	< 0.001* (*χ*^2^ = 63.2,* df* = 1)
*Pathogenic agent*												
Bacteria	1.9 (9/469)	2.5 (6/241)	3.9 (5/128)	1.0 (1/98)	0 (0/15)	0.231 (FET = 8.1)	1.3 (3/228)	0 (0/114)	0 (0/57)	5.3 (3/57)	0.001* (FET = 22.0)	0.145 (FET = 5.4)
Virus	5.5 (26/469)	7.1 (17/241)	5.5 (7/128)	9.2 (9/98)	6.7 (1/15)		3.9 (9/228)	0 (0/114)	1.8 (1/57)	14.0 (8/57)		
Parasite	89.1 (418/469)	85.9 (207/241)	83.6 (107/128)	88.8 (87/98)	86.7 (13/15)		92.5 (211/228)	96.5 (110/114)	98.2 (56/57)	78.9 (45/57)		
Other/DK	3.4 (16/469)	4.6 (11/241)	7.0 (9/128)	1.0 (1/98)	6.7 (1/15)		2.2 (5/228)	3.5 (4/114)	0 (0/57)	1.8 (1/57)		
*Same species in animals/humans?*												
Yes	74.4 (265/356)	64.7 (99/153)	52.4 (44/84)	79.7 (55/69)	NA	< 0.001* (*χ*^2^ = 12.4,* df* = 1)	81.8 (166/203)	74.0 (74/100)	89.1 (49/55)	89.6 (43/48)	0.018* (*χ*^2^ = 8.0,* df* = 2)	< 0.001* (*χ*^2^ = 13.4,* df* = 1)
*Main route of transmission*												
Arthropod bite	97.1 (442/455)	97.8 (225/230)	96.6 (115/119)	99.0 (96/97)	100 (14/14)		96.4 (217/225)	92.8 (103/111)	100 (57/57)	98.2 (56/57)		
Mosquito bite	35.3 (156/442)	43.6 (98/225)	53.9 (62/115)	29.2 (28/96)	57.1 (8/14)		26.7 (58/217)	41.7 (43/103)	8.8 (5/57)	17.9 (10/56)		
Sand fly bite	72.4 (320/442)	66.2 (149/225)	56.5 (65/115)	84.4 (81/96)	21.4 (3/14)		78.8 (171/217)	65.0 (67/103)	100 (57/57)	83.9 (47/56)		
Flea bite	7.9 (35/442)	13.3 (30/225)	17.4 (20/115)	7.3 (7/96)	21.4 (3/14)		2.3 (5/217)	4.9 (5/103)	0 (0/57)	0 (0/56)		
Tick bite	9.0 (40/442)	14.2 (32/225)	19.1 (22/115)	6.2 (6/96)	28.6 (4/14)		3.7 (8/217)	5.8 (6/103)	1.8 (1/57)	1.8 (1/56)		
Direct contact with animals	12.7 (58/455)	13.5 (31/230)	19.3 (23/119)	8.2 (8/97)	0 (0/14)		12.0 (27/225)	15.3 (17/111)	3.5 (2/57)	14.0 (8/57)		
Animal bite/scratch	7.5 (34/455)	10.4 (24/230)	13.4 (16/119)	8.2 (8/97)	0 (0/14)		4.4 (10/225)	4.5 (5/111)	1.8 (1/57)	7.0 (4/57)		
Blood transfusion	21.1 (96/455)	21.3 (49/230)	13.4 (16/119)	32.0 (31/97)	14.3 (2/14)		20.9 (47/225)	18.0 (20/111)	31.6 (18/57)	15.8 (9/57)		
Vertical	14.5 (66/455)	13.0 (30/230)	10.9 (13/119)	16.5 (16/97)	7.1 (1/14)		16.0 (36/225)	13.5 (15/111)	26.3 (15/57)	10.5 (6/57)		
Organ transplant	8.8 (40/455)	7.0 (16/230)	4.2 (5/119)	11.3 (11/97)	0 (0/14)		10.7 (24/225)	13.5 (15/111)	12.3 (7/57)	3.5 (2/57)		
DK/CR	3.8 (18/473)	5.3 (13/243)	8.5 (11/130)	1.0 (1/98)	6.7 (1/15)	0.046* (FET = 6.2)	2.2 (5/230)	4.3 (5/116)	0 (0/57)	0 (0/57)	0.515 (FET = 1.3)	0.071 (*χ*^2^ = 3.3,* df* = 1)
*Is animal leishmaniasis diagnosed in Portugal?*												
Yes	87.4 (333/381)	85.8 (188/219)	79.3 (96/121)	93.9 (92/98)	80.0 (12/15)	0.008* (FET = 9.6)	89.5 (145/162)	83.8 (88/105)	100 (57/57)	96.5 (55/57)	0.002* (FET = 12.4)	0.287 (*χ*^2^ = 1.1, * df* = 1)
*Is human leishmaniasis diagnosed in Portugal?*												
Yes	69.4 (218/314)	49.3 (73/148)	51.2 (41/80)	47.1 (32/68)	NA	0.611 (*χ*^2^ = 0.26,* df* = 1)	87.3 (145/166)	91.9 (102/111)	78.2 (43/55)	NA	0.012* (*χ*^2^ = 6.3, * df* = 1)	< 0.001* (*χ*^2^ = 53.3,* df* = 1)
*Is leishmaniasis endemic in Portugal?*												
Yes	98.6 (354/359)	99.3 (144/145)	97.6 (40/41)	100 (92/92)	100 (12/12)	0.280 (FET = 2.5)	98.1 (210/214)	97.1 (99/102)	100 (57/57)	98.2 (54/55)	0.850 (FET = 0.32)	0.349 (FET = 0.65)
*Animal species most affected*												
Dogs	98.0 (345/352)	97.0 (194/200)	97.9 (95/97)	96.7 (88/91)	91.7 (11/12)	0.372 (FET = 1.5)	99.3 (151/152)	100 (86/86)	100 (11/11)	98.2 (54/55)	0.434 (FET = 2.9)	0.146 (FET = 2.4)
Cats	19.9 (70/352)	26.5 (53/200)	37.1 (36/97)	14.3 (13/91)	33.3 (4/12)		11.3 (17/151)	15.1 (13/86)	0 (0/11)	7.3 (4/55)		
Other(s)^a^	19.6 (69/352)	20.0 (40/200)	21.6 (21/97)	20.9 (19/91)	0 (0/12)		19.2 (29/151)	20.9 (18/86)	9.1 (1/11)	18.2 (10/55)		
DK/CR	2.5 (9/361)	2.9 (6/206)	2.0 (2/99)	4.2 (4/95)	0 (0/12)	0.609 (FET = 1.5)	1.9 (3/155)	2.3 (2/88)	9.1 (1/12)	0 (0/55)	0.164 (FET = 1.8)	0.734 (FET = 0.35)
*Presentation of Leishmania infection*												
Always symptomatic	7.0 (21/301)	3.5 (5/142)	1.8 (1/55)	3.8 (3/79)	12.5 (1/8)	0.304 (FET = 4.8)	10.1 (16/159)	15.1 (8/53)	1.8 (1/57)	14.3 (7/49)	0.006* (FET = 14.4)	0.015* (*χ*^2^ = 8.3,* df* = 2)
Mostly symptomatic	68.4 (206/301)	66.2 (94/142)	60.0 (33/55)	68.4 (54/79)	87.5 (7/8)		70.4 (112/159)	58.5 (31/53)	73.7 (42/57)	79.6 (39/49)		
Mostly asymptomatic	24.6 (74/301)	30.3 (43/142)	38.2 (21/55)	27.8 (22/79)	0 (0/8)		19.5 (31/159)	26.4 (14/53)	24.6 (14/57)	6.1 (3/49)		
DK/CR	19.1 (71/372)	30.7 (63/205)	40.9 (38/93)	18.6 (18/97)	46.7 (7/15)	0.001* (*χ*^2^ = 13.0,* df* = 2)	4.8 (8/167)	0 (0/53)	0 (0/57)	14.0 (8/57)	0.006* (FET = 10.2)	< 0.001* (χ2 = 40.1,* df* = 1)
*Is there any treatment in humans?*												
Yes	68.8 (159/231)	57.4 (81/141)	70.3 (45/64)	44.1 (30/68)	66.7 (6/9)	0.008* (FET = 9.6)	86.7 (78/90)	83.3 (35/42)	NA	89.6 (43/48)	0.384 (*χ*^2^ = 0.76,* df* = 1)	< 0.001* (*χ*^2^ = 21.9,* df *= 1)
*Is there any treatment in animals?*												
Yes	67.1 (114/170)	57.3 (63/110)	NA	56.2 (54/96)	64.3 (9/14)	0.570 (*χ*^2^ = 0.32,* df* = 1)	85.0 (51/60)	NA	100 (6/6)	83.3 (45/54)	0.415 (FET = 0.58)	< 0.001* (*χ*^2^ = 13.5,* df* = 1)
*Vaccine available for dogs?*												
Yes	76.3 (190/249)	82.8 (82/99)	NA	84.1 (74/88)	72.7 (8/11)	0.346 (FET = 0.40)	72.0 (108/150)	64.7 (55/85)	100 (11/11)	77.8 (42/54)	0.068 (FET = 5.4)	0.049* (*χ*^2^ = 3.9,* df* = 1)

Values in table are presented as the percentage with the numbers in parentheses

*DK/CR* Don't know/Can't remember,* EH *Environmental Health,* EHT* Environmental Health technicians,* FET* Fisher’s exact test,* NA* not available

*Statistically significant according to Chi-square test or FET, as shown

^a^Including horses, sheep, goats, cattle, rabbits, other domestic animals, wild carnivores, other wild animals

Around 90% of participants identified the pathogenic agent as a parasite. Of those who recognized animal and human disease, approximately 75% answered that the same species of *Leishmania* infects both animals and humans. Arthropod bite was identified as the main route of transmission by 97.1% of participants; among these, sand fly bite was the predominant answer, followed by mosquito bite, although the difference between the two was less pronounced for students. More than 10% of participants answered that leishmaniasis could be transmitted by direct contact with animals. When questioned about the periods of highest phlebotomine activity, > 50% of the EHTs selected each month between May and September (inclusively); 82.2% selected dusk and 51.1% selected night. The preferred sand fly breeding grounds pointed by the EHTs were domestic animal shelters (67.4%), decomposing vegetal matter (50.0%), wild animal burrows (32.6%) and small water bodies (28.3%).

**Fig. 3:**
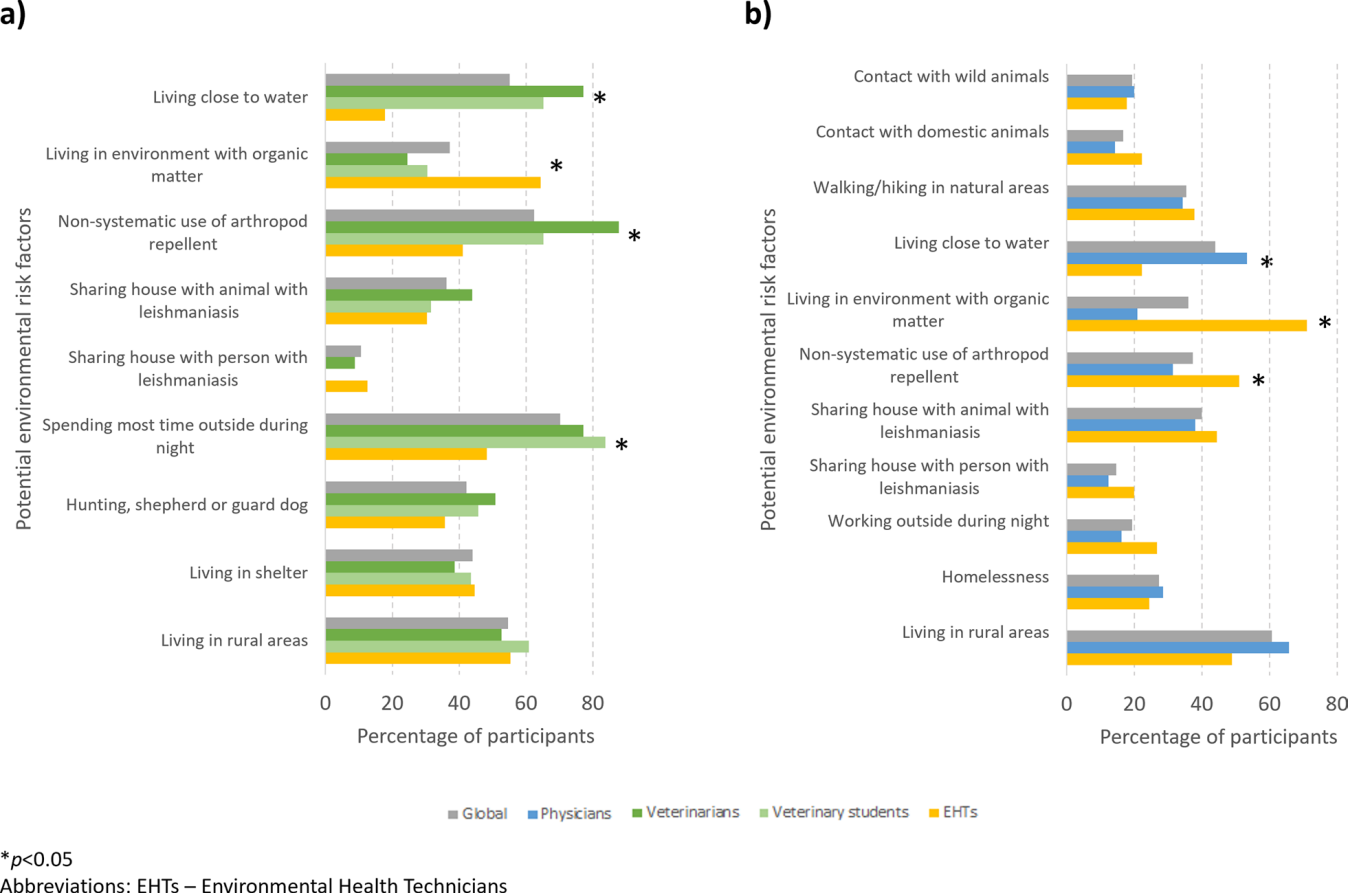
Percentage of participants, globally and by student/professional group, who selected each potential environmental risk factor for animal leishmaniasis (**a**) and human leishmaniasis (**b**). Asterisk indicates a significant difference at *P < 0.05

Individual risk factors for leishmaniasis most often selected by medical students, physicians and EHTs were HIV infection/AIDS and use of immunosuppressive drugs; physicians who had previously diagnosed VL also recognized active malignancy and solid organ transplant as important risk factors. Both veterinarians and veterinary students selected the use of immunosuppressive drugs as the highest risk factor; however, the second most often selected answer was animal breed for veterinarians and juvenile age for students. For all groups, male sex was the least frequently selected risk factor. Figure 3 shows the percentage of participants who selected each potential environmental risk factor for animal or human leishmaniasis. The three most selected environmental risk factors for animal leishmaniasis were: (i) spending most time outside during night; (ii) non-systematic use of arthropod repellent; and (iii) living close to water. Veterinary students and veterinarians selected these three risk factors significantly more often than EHTs, who selected living in an environment with organic matter significantly more often than veterinary students and veterinarians. For human leishmaniasis, living in rural areas was clearly the most selected risk factor; living close to water was significantly more selected by physicians and living in an environment with organic matter was significantly more selected by EHTs.

Animal leishmaniasis was considered to be diagnosed in Portugal by 87.4% of participants and human leishmaniasis by 69.4%. Among human health students/professionals, diagnosis of VL in Portugal was recognized more commonly than diagnosis of CL (73.9% vs 60.6%, *P* = 0.009). Almost every participant who considered leishmaniasis was diagnosed in Portugal answered that the disease was endemic. The percentage of physicians who considered the disease was imported in all or most cases was 24.1% for VL and 54.0% for CL; for medical students, however, it was 82.4% for VL and 56.2% for CL. On the other hand, 92.2% of veterinarians considered cases of CanL were all or mostly autochthonous.

Regarding animal hosts, dogs were almost universally selected as the species most affected by leishmaniasis; 19.9% of participants selected cats.

**Fig. 4:**
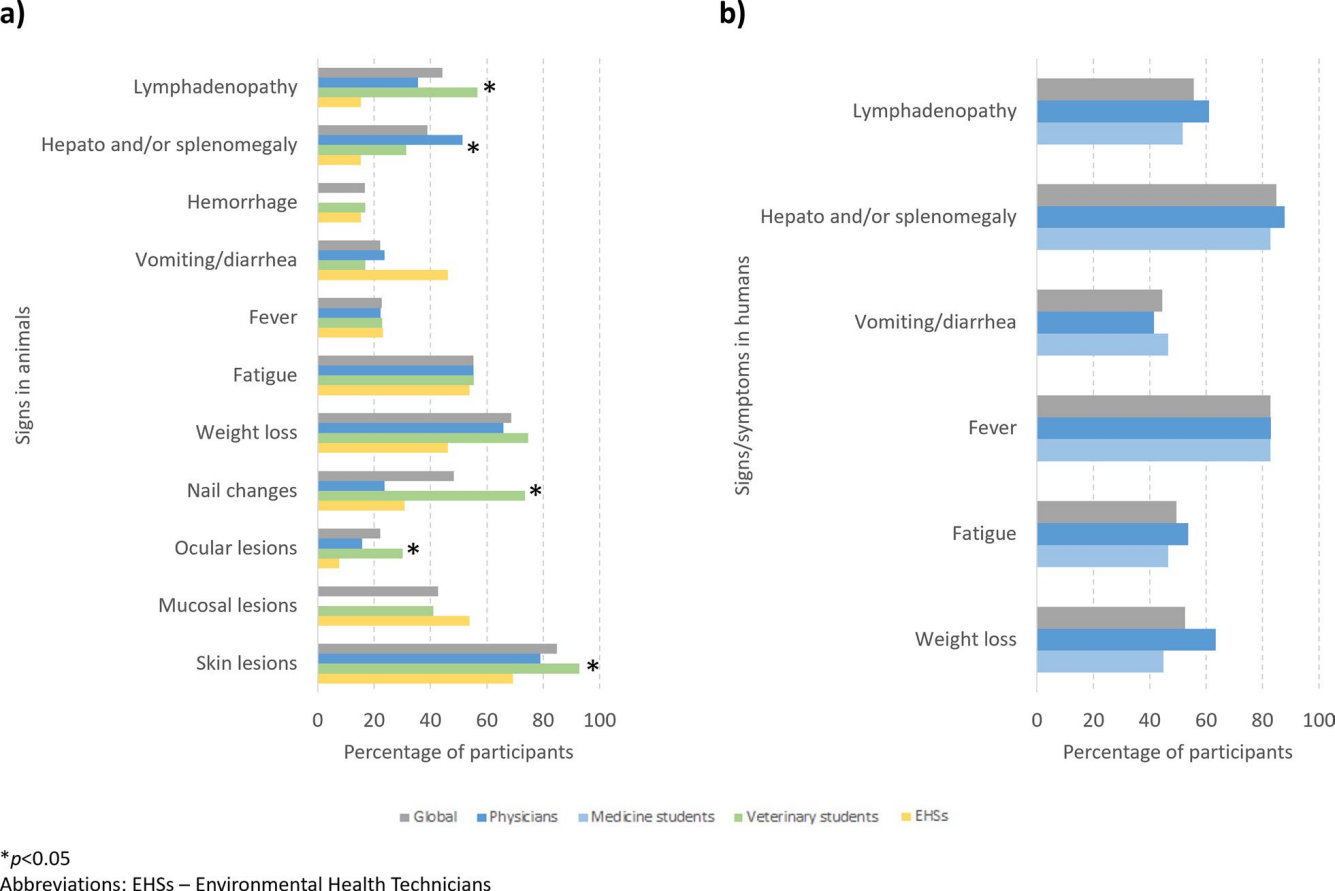
Percentage of participants, globally and by student/professional group, who selected each sign/symptom associated with animal leishmaniasis (**a**) and human leishmaniasis (**b**)

All groups considered that *Leishmania* infection was mostly symptomatic. A significant proportion of students assumed not knowing or not remembering the signs of animal leishmaniasis (27.6% of medical students, 13.5% of veterinary students and 7.1% of EHSs). Among those participants who reported knowing the signs/symptoms of *Leishmania* infection, the percentage who selected each sign/symptom associated with animal or human leishmaniasis is shown in Fig. 4. In animal leishmaniasis, skin lesions, weight loss and fatigue were the most selected signs (no significant difference in percentage between groups, except for skin lesions, being less selected by EHSs); nail changes, ocular lesions and lymphadenopathy were significantly more recognized by veterinary students. In human leishmaniasis, hepato- and/or splenomegaly and fever were the most selected signs of disease; no significant difference in percentage was seen for any of the signs/symptoms analyzed between medical students and physicians.

Regarding the diagnosis of leishmaniasis, both medical students and physicians who had never seen cases of leishmaniasis considered blood to be the type of sample most used (selected by 65.6% of students and 81.6% of physicians); as the second most sample type used, physicians considered bone marrow (47.4%) and medical students considered lymph node (26.2%). Blood was also the type of sample most selected by veterinary students (89.8%), followed by lymph node (30.7%).

Around 15% of veterinary and medical students assumed that they did not know the diagnostic techniques most often used. Among those who did know, the techniques most selected were serology (50.6%) and microscopy (33.7%) for veterinary students, and microscopy (61.1%) for medicine students; in contrast, 78.4% of physicians who had never seen cases of leishmaniasis selected PCR and 64.9% microscopy.

Leishmaniasis was considered to be treatable in humans by 68.8% of participants and treatable in animals by 67.1%. In addition, 76.3% of participants recognized that a vaccine against leishmaniasis was available for dogs. The declaration of cases to Public or Animal Health authorities was considered to be mandatory by 43.9% of veterinarians for CanL and by 96.9% and 66.2% of physicians for VL and CL, respectively.

### Results on perceptions

Answers to individual questions on perceptions are summarized in Table 3. Most veterinarians (77.4%) considered that there were > 50 cases of leishmaniasis diagnosed in their region of work. Among physicians who had previously diagnosed leishmaniasis, 86.8% considered that there was a “low” risk of a person developing the disease in their area of work. Additionally, the number of VL cases diagnosed in their region in the last 10 years was considered to be decreasing (48.7%) or stable (41.0%). For CL, 90.0% considered the number of cases to be stable. In contrast, 56.5% of veterinarians who had previously diagnosed leishmaniasis considered the number of cases to have increased in the last 10 years.

**Table 3:** Answers to the questions on perceptions, globally and by student/professional group

Perceptions	All	Students					Professionals					*P*-value global
		Global	Medicine	Veterinary science	EH	*P*-value	Global	Physicians	Veterinarians	EHTs	*P*-value	
*How important is it to include leishmaniasis in the training of (0–4)?*												
Veterinary students	3.71	3.70	3.64	3.76	3.92	0.063 (*H* = 5.5,* df* = 2)	3.71	3.53	3.88	3.86	< 0.001* (*H* = 25.6,* df* = 2)	0.711 (*H* = 0.1,* df* = 1)
Medical students	3.16	3.01	2.80	3.25	3.44	< 0.001* (*H* = 14.1,* df* = 2)	3.27	2.93	3.55	3.75	< 0.001* (*H* = 48.7,* df* = 2)	0.002* (*H* = 9.7,* df* = 1)
EH students	3.61				2.87					3.80		< 0.001* (H = 15.7,* df* = 1)
Professionals of respective group								3.25	3.73	3.77	< 0.001* (*H* = 35.1,* df* = 2)	
*How important do you think is the collaboration between physicians, veterinarians and EHT to eliminate leishmaniasis (0–3)?*												
	2.89	2.92	2.88	2.96	3.00	0.150 (*H* = 3.8,* df* = 2)	2.87	2.82	2.87	2.98	0.026* (*H* = 7.3,* d*f = 2)	0.154 (*H* = 2.0,* df* = 1)

Values in table are the mean values for each group

*EH* Environmental Health,* EHT* Environmental Health technicians

*Statistically significant according to the Kruskal–Wallis H-test

Inclusion of leishmaniasis in the veterinary curriculum was considered similarly important by students and professionals and among different subgroups of students; among professionals, however, physicians considered the inclusion of leishmaniasis as less important. All groups considered it to be more important to include leishmaniasis in the veterinary curriculum than in the human medicine curriculum; it was considered to be less important by more students than health professionals, and to be less important by students/professionals in the human health field. Inclusion of leishmaniasis in the academic program was considered to be more important by EHSs than by professionals. Regarding the inclusion of leishmaniasis in the training of professionals, physicians assessed as a group considered it to be less relevant for themselves. In terms of medical specialties for which training on leishmaniasis was considered to be more important, 95.5% selected Infectious Diseases, 87.5% Internal Medicine, 83.0% Pediatrics, 74.1% Dermatology, 56.2% Anatomopathology and 19.6% Others.

The importance of collaboration between sectors was rated equally by students and professionals (Kruskal–Wallis test,* H* = 2.0,* df* = 1, *P* = 0.154); among professionals, importance was rated in descending order as: EHTs, veterinarians, physicians (Kruskal–Wallis test,* H* = 7.3,* df* = 2, *P* = 0.026). The creation of national guidelines for the diagnosis and management of leishmaniasis was considered to be “very important” by 84.0% of physicians for VL and 71.4% for CL. The implementation of a national structured plan to control leishmaniasis was considered to be "very important" by 89.6% of veterinarians and 87.5% of EHTs. Additionally, only 38.6% of veterinarians and 12.5% of physicians were satisfied with the information on animal or human leishmaniasis that appeared on official platforms, respectively (the remaining participants were not or had no opinion). Similarly, only 42.2% of veterinary students and 15.8% of medicine students were “very satisfied” with the quantity and quality of information presented in their courses.

**Fig. 5:**
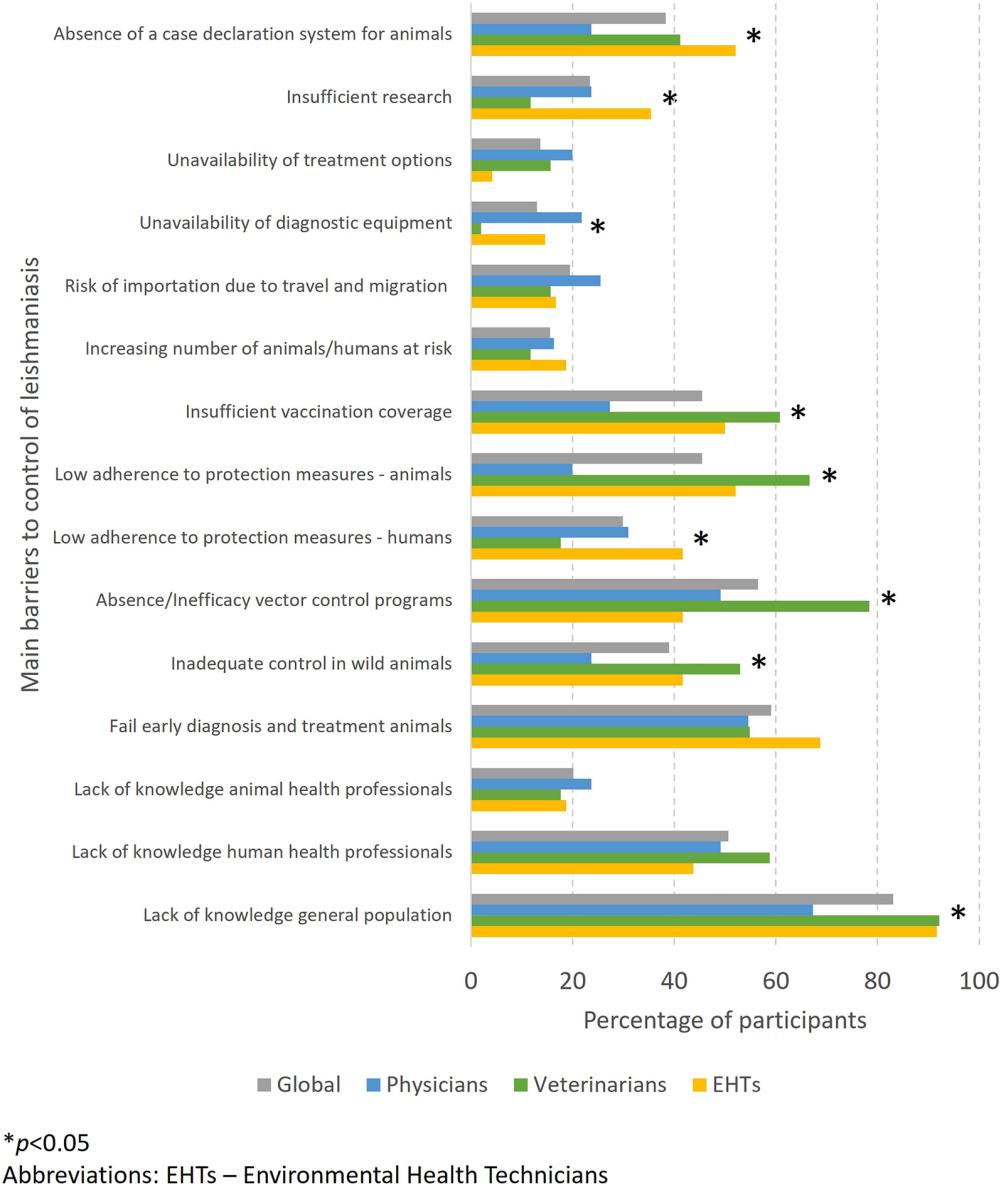
Percentage of participants, globally and by student/professional group, who selected each potential main barrier to the control of leishmaniasis. Asterisk indicates a significant difference at *P < 0.05

The main barriers pointed by professionals to the control of leishmaniasis were (note: options selected by > 50% of respondents): lack of knowledge in the general population, failure in early diagnosis and treatment of diseased animals, absence/inefficacy of vector control programs and lack of knowledge by human health professionals. Significant differences were reported for some of these barriers, as shown in Fig. 5. Low adherence to protection measures in animals, insufficient vaccination coverage and inadequate control in wild animals were the most common barriers considered by veterinarians, while absence of a case declaration system for animals, low adherence to protection measures in humans and insufficient research were pointed out mostly by EHTs. Unavailability of diagnostic equipment was the most common barrier considered by physicians.

**Fig. 6:**
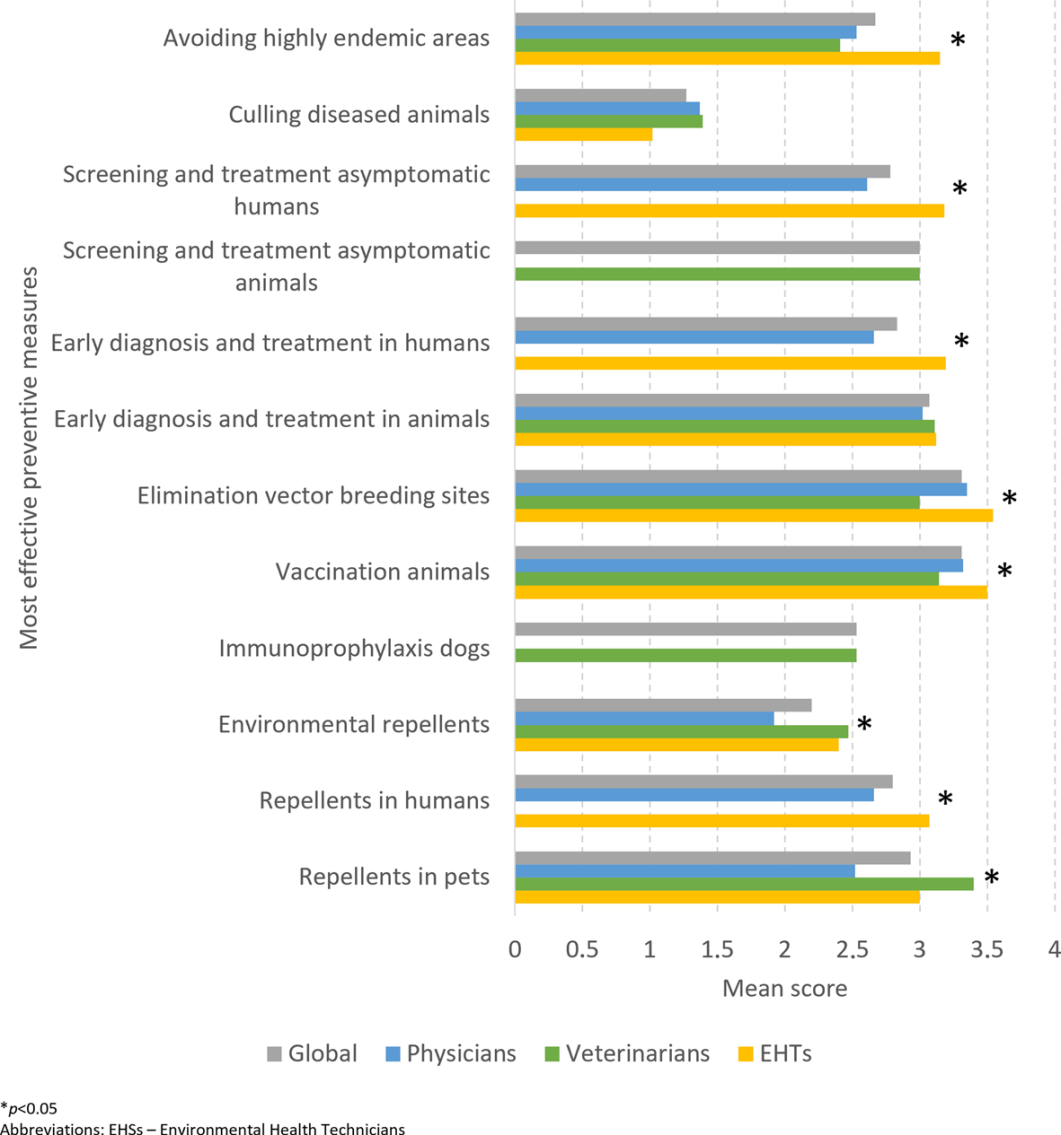
Mean score for each possibly most effective measure in leishmaniasis control, globally and by student/professional group. Asterisk indicates a significant difference at *P < 0.05

The mean scores for effective measures in leishmaniasis control is shown in Fig. 6. Overall, vaccination of animals, elimination of vector breeding sites, early diagnosis and treatment of diseased animals and use of repellents in pets were considered to be the most effective measures. Most measures evaluated were scored significantly differently between groups: use of repellent in pets and use of environmental repellents were scored higher by veterinarians; vaccination of animals, elimination of vector breeding sites and avoiding highly endemic areas were scored higher by EHTs. Early diagnosis and treatment in animals and culling of diseased animals were not scored significantly differently.

### Results on general practices

Answers to individual questions on general practices are summarized in Table 4. Seeing cases of leishmaniasis in course/work was more common for professionals than for students (Chi-square test,* χ*^2^ = 76.5,* df* = 1, *p* < 0.001) and in the animal health field than in the human (Chi-square test, * χ*^2^ = 27.5,* df* = 1, *p* < 0.001).

**Table 4:** Answers to general practices questions, globally and by student/professional group

General practices questions	All	Students					Professionals					*p*-value global
		Global	Medicine	Veterinary science	EH	*P*-value	Global	Physicians	Veterinarians	EHTs	*P*-value	
*Seen cases in course/work?*												
Yes	39.1 (127/325)	13.8 (21/152)	0 (0/71)	25.9 (21/81)	NA	< 0.001* (FET = 18.6)	61.3 (106/173)	48.3 (56/116)	89.5 (51/57)	NA	< 0.001* (*χ*^2^ = 27.5,* df* = 1)	< 0.001* (*χ*^2^ = 76.5, * df* = 1)
*Repellent use in outdoor activities during dusk/night*												
Always	4.7 (19/403)	5.1 (11/215)	6.6 (8/122)	2.6 (2/78)	6.7 (1/15)	0.772 (FET = 1.8)	4.3 (8/188)	3.2 (3/95)	2.1 (1/47)	8.7 (4/46)	0.471 (FET = 3.5)	0.020* (*χ*^2^ = 7.9, * df* = 2)
Sometimes	32.8 (132/403)	38.6 (83/215)	36.9 (45/122)	41.0 (32/78)	40.0 (6/15)		26.1 (49/188)	24.2 (23/95)	29.8 (14/47)	26.1 (12/46)		
Never	62.5 (252/403)	56.3 (121/215)	56.6 (69/122)	56.4 (44/78)	53.3 (8/15)		69.7 (131/188)	72.6 (69/95)	68.1 (32/47)	65.2 (30/46)		
Not applicable	16.7 (81/484)	15.0 (38/253)	12.9 (18/140)	19.6 (19/97)	6.2 (1/16)		18.6 (43/231)	17.4 (20/115)	17.5 (10/57)	22.0 (13/59)		
*Nets in doors/windows*												
All	4.1 (20/484)	5.9 (15/253)	6.4 (9/140)	5.2 (5/97)	6.2 (1/16)	0.974 (FET = 0.49)	2.2 (5/231)	3.5 (4/115)	0 (0/57)	1.7 (1/59)	0.405 (FET = 4.0)	0.051 (*χ*^2^ = 6.0, * df* = 2)
Some	15.3 (74/484)	17.0 (43/253)	15.7 (22/140)	18.6 (18/97)	18.8 (3/16)		13.4 (31/231)	10.4 (12/115)	12.3 (7/57)	20.3 (12/59)		
No	80.6 (390/484)	77.1 (195/253)	77.9 (109/140)	76.3 (74/97)	75.0 (12/16)		84.4 (195/231)	86.1 (99/115)	87.7 (50/57)	78.0 (46/59)		
*Pet ownership*												
Dog(s)	47.3 (229/484)	57.7 (146/253)	48.9 (68/139)	72.4 (71/98)	43.8 (7/16)	< 0.001* (FET = 36.8)	35.9 (83/231)	28.7 (33/115)	52.6 (30/57)	33.9 (20/59)	< 0.001* (*χ*^2^ = 36.8, * df* = 4)	< 0.001* (*χ*^2^ = 23.0, * df* = 2)
Other(s)	25.8 (125/484)	20.9 (53/253)	16.5 (23/139)	24.5 (24/98)	37.5 (6/16)		31.2 (72/231)	23.5 (27/115)	45.6 (26/57)	32.2 (19/59)		
No	26.9 (130/484)	21.3 (54/253)	34.5 (48/139)	3.1 (3/98)	18.7 (3/16)		32.9 (76/231)	47.8 (55/115)	1.8 (1/57)	33.9 (20/59)		
*Dog(s) spend(s) time outdoors during dusk/night?*												
Yes	72.5 (166/229)	77.4 (113/146)	83.8 (57/68)	73.2 (52/71)	57.1 (4/7)	0.139 (FET = 3.9)	63.9 (53/83)	75.8 (25/33)	50.0 (15/30)	65.0 (13/20)	0.104 (*χ*^2^ = 4.5, * df* = 2)	0.027* (*χ*^2^ = 4.9, * df* = 1)
*Repellent use in dog(s)*												
Yes	78.5 (179/228)	77.2 (112/145)	80.6 (54/67)	74.6 (53/71)	71.4 (5/7)	0.659 (FET = 0.84)	80.7 (67/83)	69.7 (23/33)	90.0 (27/30)	85.0 (17/20)	0.107 (FET = 4.5)	0.538 (*χ*^2^ = 0.38, * df* = 1)
All year round	78.8 (141/179)	82.1 (92/112)	81.5 (44/54)	81.1 (43/53)	100 (5/5)	0.730 (FET = 0.63)	73.1 (49/67)	65.2 (15/23)	81.5 (22/27)	70.6 (12/17)	0.417 (*χ*^2^ = 1.7, * df* = 2)	0.154 (*χ*^2^ = 2.0, * df* = 1)
Some months	21.2 (38/179)	17.9 (20/112)	18.5 (10/54)	18.9 (10/53)	0 (0/5)		26.9 (18/67)	34.8 (8/23)	18.5 (5/27)	29.4 (5/17)		
Spot-on	57.4 (103/179)	59.8 (67/112)	61.1 (33/54)	54.7 (29/53)	100 (5/5)		53.7 (36/67)	65.2 (15/23)	40.7 (11/27)	58.8 (10/17)		
Collar	54.7 (98/179)	57.1 (64/112)	51.9 (28/54)	64.2 (34/53)	40.0 (2/5)		50.7 (34/67)	47.8 (11/23)	63.0 (17/27)	35.3 (6/17)		
Pills	38.0 (68/179)	39.3 (44/112)	38.9 (21/54)	43.4 (23/53)	0 (0/5)		35.8 (24/67)	47.8 (11/23)	33.3 (9/27)	23.5 (4/17)		
Shampoo	8.4 (15/179)	8.0 (9/112)	7.4 (4/54)	7.5 (4/53)	20.0 (1/5)		9.0 (6/67)	21.7 (5/23)	0 (0/27)	5.9 (1/17)		
Spray	5.0 (9/179)	5.4 (6/112)	3.7 (2/54)	7.5 (4/53)	0 (0/5)		4.5 (3/67)	8.7 (2/23)	0 (0/27)	5.9 (1/17)		
*Vaccination of dog(s)*												
Every year	42.4 (73/172)	42.4 (56/132)	41.2 (28/68)	45.6 (26/57)	28.6 (2/7)	0.660 (FET = 0.83)	42.5 (17/40)	47.6 (10/21)	25.0 (1/4)	40.0 (6/15)	0.682 (FET = 0.76)	0.993 (*χ*^2^ < 0.01, * df* = 1)
Some years/No/DK/CR	57.6 (99/172)	57.6 (76/132)	58.8 (40/68)	54.4 (31/57)	71.4 (5/7)		57.5 (23/40)	52.4 (11/21)	75.0 (3/4)	60.0 (9/15)		
*Veterinary observation of dog(s)*												
At least once a year	91.7 (209/228)	89.0 (129/145)	88.1 (59/67)	90.1 (64/71)	85.7 (6/7)	0.891 (FET = 0.23)	96.4 (80/83)	97.0 (32/33)	100 (30/30)	90.0 (18/20)	0.452 (FET = 1.6)	0.051 (* χ*^2^ = 3.8, * df* = 1)
Less than once a year	8.3 (19/228)	11.0 (16/145)	11.9 (8/67)	9.9 (7/71)	14.3 (1/7)		3.6 (3/83)	3.0 (1/33)	0 (0/30)	10.0 (2/20)		

Values in table are presented as the percentage with the numbers in parentheses

*DK/CR* Don't Know/Can't remember, *EH* Environmental Health,* EHT* Environmental Health technicians,* FET* Fisher’s exact test

*Statistically significant according to the Chi-square test or FET, as shown

Most participants reported "Never" to using arthropod repellent during outdoor activities at dusk/night, with the proportion of "Never' responses being significantly higher in professionals (Chi-square test, *χ*^2^ = 7.9,* df* = 2, *P* = 0.020). Most participants mentioned having "No" nets in doors/windows, and this proportion was similar between groups (Chi-square test, *χ*^2^ = 6.0, * df* = 2, *P* = 0.051). Approximately half of the participants (47.3%) were dog owners, although dog ownership was significantly more common among students (57.7 vs 35.9%) and professionals in the animal health field (Fisher’s exact test, 36.8, *P* < 0.001). Most participants reported that their dog(s) spent time outside between dusk and dawn, although this response was higher for students (Chi-square test, *χ*^2^ = 4.9,* df* = 1, *p* = 0.027). Of the dog owners who mentioned that their dogs spent time outdoors during the night, 77.1% selected garden/yard, 30.1% street/road, 9.6% forest/bush and 5.4% other.

The use of arthropod repellent on dog(s) was reported by > 75% of participants and was year-round for most dogs (78.8%), with no significant differences between groups. In general, spot-on treatments were the mostly commonly used arthropod repellent method used by both students and professionals, followed by collars and pills. Among veterinarians and veterinary students, however, collars were preferred over spot-on treatments. Approximately 40% of the participants reported vaccinating their dog(s) "every year," with similar proportions among groups (Chi-square test, *χ*^2^ < 0.01,* df* = 1, *p* = 0.993). Over 90% of participants mentioned taking their dog(s) for veterinary observation "at least once a year" and this was similar between groups (Chi-square test, *χ*^2^ = 3.8,* df* = 1, *p* = 0.051).

### Results on professional practices

Answers to individual questions on professional practices are summarized in Table 5. Among professionals who had seen leishmaniasis, 77.1% of veterinarians and 11.3% of physicians had seen > 10 cases. Of physicians who reported having been involved in diagnosing/treating patients with leishmaniasis, 75.0% mentioned only VL, 3.6% only CL and 21.4% both.

**Table 5:** Answers to questions on professional practices, globally and by professional group

Questions on professional practices	Veterinarians	Physicians	*P*-value
	CanL	VL	
*Number of cases seen, % (n)*			
1–5	14.6 (7/48)	75.5 (40/53)	< 0.001* (*χ*^2^ = 46.2,* df* = 2)
6–10	8.3 (4/48)	13.2 (7/53)	
11–50	39.6 (19/48)	11.3 (6/53)	
> 50	37.5 (18/48)		
*Type of sample used for diagnosis (0–3)*^a^			
Bone marrow biopsy/aspirate	0.49	2.22	< 0.001* (*U* = 298.0)
Liver biopsy/aspirate		0.85	
Lymph node biopsy/aspirate	1.00	1.12	0.485 (*U* = 1129.5)
Spleen biopsy/aspirate	0.20	0.43	0.074 (*U* = 1005.5)
Skin	0.78		
Blood	2.84	2.18	< 0.001* (*U* = 808.5)
*Type of laboratory exam used for diagnosis (0–3)*^a^			
Culture	0.24	0.96	< 0.001* (*U* = 722.0)
Microscopy of smear/histology	0.78	2.08	< 0.001* (*U* = 501.5)
PCR	1.32	2.19	< 0.001* (*U* = 647.5)
Quantitative serology	2.53	1.77	< 0.001* (*U* = 623.5)
Qualitative serology	2.23		
*Drug used for treatment (0–4)*^b^			
Miltefosine	1.75	1.11	< 0.001* (*U* = 824.5)
MA/Pentavalent antimonials	2.02	0.80	< 0.001* (*U* = 536.0)
Allopurinol	3.30	NA	
Allopurinol + MA	2.26	NA	
Allopurinol + miltefosine	1.90	NA	
Liposomal amphotericin B	NA	3.32	
Paromomycin	NA	0.26	
Amphotericin B deoxycholate	NA	0.24	
Pentamidine	NA	0.12	
Recommended individual protective measures to patients, %, (*n*)			
Often/always	93.0 (53/57)	8.1 (8/99)	< 0.001* (*χ*^2^ = 109.5,* df* = 1)

*CanL* Canine leishmaniasis,* MA* meglumine antimoniate,* NA* not available,* VL* visceral leishmaniasis

*Statistically significant according to the Mann-Whitney U-test or Chi-square test, as shown

^a^ Answer options were provided in a scale: "0 - Never", "1 - Sometimes", "2 - Often" and "3 - Always". Values in the lines below are the mean values for each group

^b^ Answer options were provided in a scale: "0 - Never", "1 - In some cases", "2 - About half of the cases", "3 - In most cases" and "4 - In all cases". Values in the lines below are the mean values for each group

The most commonly used samples for diagnosing VL were bone marrow biopsy/aspirate and blood. PCR was the most common technique used for diagnosis, followed by microscopy. The factors reported to most influence the choice of diagnostic method for VL were availability (72.9%), sensitivity (72.9%), specificity (64.6%) and speed (54.2%) of test; need to send a sample to an external laboratory (16.7%); and cost (2.1%).

Liposomal amphotericin B (LAmB) was the drug reported as most often used to treat VL, followed by miltefosine. LAmB was considered to be more effective than miltefosine, and both drugs were suggested as being more effective in immunocompetent than immunosuppressed patients. Only 6/44 physicians reported ever using amphotericin B deoxycolate, 7/45 paromomycin and 4/42 pentamidine. The factors reported out to most influence the choice of treatment for VL were availability (83.0%), side effects (55.3%), degree of immunosuppression (40.4%), formulation (27.7%) and cost (10.6%). In the case of failure/relapse, 58.6% of physicians reported initiating treatment with combination of drugs and 24.1% reported continuing with the same drug for a longer duration.

Only 14/116 physicians reported having seen cases of CL (all but one mentioned 1–5 cases). Of these, 61.5% reported having seen CL cases resulting from infection in Latin America, 38.5% from infection in southern Europe, 30.8% from infection in the Middle East and 15.4% from infection in North Africa. The most frequently observed type of lesion was an ulcer, and 86.7% of physicians reported obtaining samples by excisional biopsy. The techniques most often used for CL diagnosis were (in descending order): microscopy, PCR, serology and culture. Only 20% of respondents answered that they “Always” or “Often” identified the infecting species of *Leishmania* in their cases. Among the species identified, *L. infantum* was mentioned most (*n* = 6). The most commonly used treatment strategies for CL were watch and wait, LAmB, miltefosine and intralesional antimonial; none of these strategies was frequently used by > 30% of clinicians. Factors most selected as influencing the choice of treatment were availability (70%), number (60%) and location of lesions (50%) and side effects (50%). Among clinicians, 58.3% considered CL treatment to be “moderately effective.”

For CanL, serology was the technique reported to be most used for diagnosis. Most veterinarians (66.7%) said they “always” recommended treatment of *Leishmania* infection in dogs while 25.5% initiated treatment only if dogs were symptomatic, regardless of severity. Allopurinol alone was the treatment regimen most often reported, followed by a combination of allopurinol + meglumine antimoniate (MA) and MA alone. Allopurinol + MA was considered to be more effective than single drug treatments, both in moderate/mild and in severe disease. Seeing cases of leishmaniasis in other animals was reported by 14.0% (8/57) of veterinarians, all of whom mentioned cats (1–2 cases each) and one mentioned a horse.

Most veterinarians (93.0%) but only 8.1% of physicians recommended individual protection measures to patients.

### Scoring KPP and associations with sociodemographic factors

The distribution of individual K, Per and Pra scores is shown in Additional file 4: Figure S3. Median K and Per scores were higher in professionals than in students. Among students, median K and Per scores were significantly higher in veterinary students, while among professionals, they were significantly higher in veterinarians and EHTs. Median Pra scores were not significantly different between groups and subgroups. Factors associated with higher K score in the univariate analysis were age > 20 years (for students) or > 30 years (for professionals), higher academic year of study, more years of professional experience, Infectious Diseases specialty, having seen cases at work and specialist level. In the multivariate analysis, however, for students, only higher academic year of study was associated with a K score higher than the median K score (OR 3.49, 95% CI 1.48–8.21, *P* = 0.004) and for professionals, only for physicians was having seen cases of leishmaniasis associated with a K score higher than the median K score (OR 14.23, 95% CI 3.79–53.45, *P* < 0.001). Factors associated with higher Per score in the univariate analysis were professional status, age > 25 years, residing outside the Centro or LVT regions, no dog ownership and K score above the median K score (> 6.5). However, in the multivariate analysis, no dog ownership (OR 2.19, 95% CI 1.46–3.27, *P* < 0.001) and K score above the median K score (> 6.5) (OR 4.69, 95% CI 3.03–7.28, *p* < 0.001) were the only factors associated with a higher than median Per score globally. Factors associated with higher Pra score in the univariate analysis were age > 30 years old and living outside the Norte region; in the multivariate analysis, both factors remained significant (OR 1.88, 95% CI 1.08–3.26, *P* = 0.025; and OR 1.56, 95% CI 1.03–2.34, *P* = 0.035, respectively) (Table 6).

**Table 6:** Potential factors for knowledge (students and professionals), perception and practices scores above the respective median score, according to logistic regression models to estimate crude and adjusted odds ratio values

KPP	Potential risk factors	Univariate logistic regression model			Multivariate logistic regression model		
		Percentage in sample	Crude OR	95% CI	Adjusted OR	95% CI	*P*-value
K > 6.5 (students)^a^	Female gender	83.3	1.37	0.62–3.03	1.65	0.71–3.80	0.242
	Age > 20 years	58.3	2.67	1.43–4.97	1.37	0.62–3.00	0.434
	Year of study > 2nd	60.9	4.23	2.08–8.61	3.49	1.48–8.21	0.004*
	No ownership of dog(s)	50.4	1.54	0.87–2.70	1.49	0.82–2.73	0.193
	Constant				0.036		< 0.001*
	Hosmer and Lemeshow test				Sig. = 0.975		

	Potential risk factors	Univariate logistic regression model			Multivariate logistic regression model		
		Percentage in Sample	Crude OR	95% CI	Adjusted OR	95% CI	*p*-value
K > 6.5 (professionals)^b^	Male gender	28.4	3.01	1.25–7.25	1.48	0.43–5.12	0.534
	Age > 30 years	72.4	3.76	1.58–8.97	1.12	0.14–8.70	0.913
	Nyp experience ≥ 10	47.4	2.77	1.30–5.91	1.93	0.47–7.87	0.360
	ID specialty	23.1	14.88	3.28–67.63	2.05	0.34–12.26	0.432
	Seen cases of leishmaniasis	48.3	14.36	5.76–35.83	14.23	3.79–53.45	< 0.001*
	Specialist level	64.7	5.40	2.26–12.89	2.47	0.33–18.22	0.376
	No ownership of dog(s)	51.7	2.54	1.20–5.41	2.57	0.87–7.56	0.087
	Constant				0.043		0.092
	Hosmer and Lemeshow test				Sig. = 0.419		

	Potential risk factors	Univariate logistic regression model			Multivariate logistic regression model		
		Percentage in sample	Crude OR	95% CI	Adjusted OR	95% CI	*p*-value
Per score > 9 (global)^c^	Professional	47.7	2.20	1.53–3.17	1.61	0.74–3.49	0.226
	Female gender	80.2	1.27	0.80–2.00	1.57	0.94–2.61	0.085
	Age > 25 years	54.2	1.98	1.37–2.86	1.42	0.65–3.13	0.381
	Residing outside Centro/LVT	38.3	1.62	1.12–2.35	1.51	0.99–2.28	0.051
	No ownership of dogs	47.4	2.23	1.54–3.23	2.19	1.46–3.27	< 0.001*
	K score > 6.5	45.5	5.11	3.47–7.53	4.69	3.03–7.28	< 0.001*
	Constant				0.452		0.116
	Hosmer and Lemeshow test				Sig. = 0.470		

	Potential risk factors	Univariate logistic regression model			Multivariate logistic regression model		
		Percentage in sample	Crude OR	95% CI	Adjusted OR	95% CI	*p*-value
Pra score > 1.5 (global)^d^	Professional	47.7	1.10	0.76–1.57	1.45	0.84–2.51	0.180
	Age > 30 years	37.4	1.45	1.01–2.10	1.88	1.08–3.26	0.025*
	Residing outside Norte	70.4	1.54	1.03–2.32	1.56	1.03–2.34	0.035*
	K score > 6.5	45.5	1.24	0.87–1.78	1.17	0.78–1.76	0.439
	Constant				0.235		0.003
	Hosmer and Lemeshow test				Sig. = 0.934		

*CI *Confidence interval,* ID* Infectious Diseases (speciality),* K* knowledge,* LVT* Lisboa e Vale do Tejo,* Nyp* number of years of professional,* OR* odds ratio,* Pra* Practices, *Per* Perceptions

* Statistically significant according to multivariate analysis

^a^Reference categories: male gender; age ≤ 20 years; first or second year of study;; ownership of dogs

^b^Reference categories: female gender; age ≤ 30 years; Nyp experience < 10; non-ID specialty; not previously seen cases of leishmaniasis; trainee level; ownership of dogs

^c^Reference categories: student; male gender; age ≤ 25 years; residing in Centro or LVT region; ownership of dogs; K score ≤ 6.5

^d^Reference categories: student; age ≤ 30 years; residing in Norte region; K score ≤ 6.5

## Discussion

This study represents the first national study of knowledge, perceptions and practices (KPP) on leishmaniasis in health students and professionals, including veterinarians. At a global level, few studies have addressed the human medicine and environmental health fields, although the zoonotic and vector-borne nature of *Leishmania* infection implies that all these fields are actively involved in its management and treatment. In the present study, not only had > 95% of students and professionals heard previously of leishmaniasis, but also the majority acknowledged it as a zoonosis, although the majority was lower in the human medicine side. Comparisons of different student categories should be seen in the light of different distribution of students by year of study; similarly, professionals of different fields were not distributed equally by years of professional experience.

Leishmaniasis seems to be consistently included in the courses in the three groups, especially in theoretical classes; it is possible that the disease is a focus of study earlier in the veterinary than in the medical curriculum. In the professional context, in addition to the direct observation of cases of disease, workshops, congresses, courses and conversations with colleagues were most often mentioned as the sources of information on leishmaniasis, highlighting the importance of continuous education and informal and peer education. Approximately half of the participants reported having heard about leishmaniasis outside of their work, with particular sources being television advertisements and conversation with veterinarians on animal leishmaniasis and via an internet search for human leishmaniasis. Although no studies in Europe have previously addressed the non-occupational sources of information on leishmaniasis, it is likely that television plays an important role via pesticide repellent advertisements for pets; however, human disease is not addressed in this platform and much less disseminated in all communication media (all less selected for human vs animal leishmaniasis).

Even though arthropod bite was correctly identified as a main route of transmission by almost every participant, mosquitoes were commonly pointed as the arthropod vector. This could explain why many participants considered living close to water to be a relevant environmental risk factor for both animal and human leishmaniasis and why only EHTs more often pointed to organic matter as a relevant risk factor. This lack of knowledge on the vector could lead to inefficient/incorrect counseling of animal owners and at risk human groups on specific prevention strategies against phlebotomine sand flies, such as no accumulation of decaying organic matter [42]; in other endemic settings, such as in areas of Brazil, veterinarians systematically recommend keeping the domestic environment free from organic matter [43]. It should be notes that direct contact with infected animals was also considered to be an important route of transmission by > 10% of participants, similarly by students and professionals, although this route has rarely been documented, and then only in dogs [44], possibly leading to inaccurate information being provided to dog/animal owners regarding their personal risk. EHTs seem to be adequately informed about periods of activity and breeding grounds of sand flies according to current knowledge on their biology [45].

Physicians and medical students referred to HIV infection/AIDS and use of immunosuppressive drugs as the most significant individual host risk factors, which is in accordance with the literature, which suggests increased risk of progression to disease in persons with HIV infection/AIDS [46], and with national data revealing that 51.8% of persons with VL diagnosed between 1999 and 2009 were coinfected with HIV and 6.5% had other immunosuppressive condition [5]. Although male sex was the least frequently selected risk factor among our respondents, studies in South Asia suggest that it as a biological risk factor, regardless of sociocultural differences based in gender [47].

The participants in our questionnaire survey considered that animal leishmaniasis was more often diagnosed in Portugal than human leishmaniasis, likely reflecting the higher incidence of the former disease and the high seroprevalence in the canine population (7). CanL and VL cases were considered to be mostly autochthonous by professionals; in available data, 76.4% of cases of VL were assumed to be autochthonous, since they were living in known endemic foci in Portugal [5]. Students were unaware of this.

Information on the situation of CL is scarce in Portugal since notification of the disease is not mandatory, while in other southern European countries it has been reported that approximately one half of CL cases seem to be imported (in Spain [48] and France [49]). In the present study, clinicians were polarized between either mostly imported or mostly autochthonous.

Asymptomatic infection has been extensively described as the most common result of exposure to *Leishmania* parasites, but only approximately 25% of students and professionals were aware of this fact, although it could be an important consideration for interpretation of positive serological results in sick individuals in endemic areas, where it could represent an incidental finding [2]. On the other hand, asymptomatic, latent infections represent both an individual and public health problem since they can reactivate and progress to overt disease in specific settings, such as in the context of iatrogenic/pharmacologic immunosuppression (in transplant, autoimmune diseases, etc.) and older age, with an aged population representing an increasing share of the population [50]. Additionally undetected and/or neglected asymptomatic infections could compromise disease control in Portugal, as it is being increasingly recognized that asymptomatic individuals can transmit the parasite to phlebotomine sand flies, especially when immunosuppressed [51].

Common signs of animal leishmaniasis (skin lesions, weight loss, fatigue) were correctly identified by most students and professionals not active in diagnosing CanL [52], although other common signs were specifically mentioned more frequently by veterinary students, such as nail and ocular changes. For human leishmaniasis, hepatosplenomegaly and fever were more often chosen by the participants, consistent with case series of VL [53].

Regarding diagnosis, students and physicians who had never seen cases of VL more often chose blood as the sample type for testing; however, bone marrow seems to be preferentially used in Portugal (5). Due to the low incidence of the disease, most physicians have never seen any case of VL, but this could change in the future, raising the possibility of a gap in knowledge of locally available diagnostic protocols and techniques. Mandatory declaration of VL cases was correctly indicated by most physicians, possibly suggesting that underreporting (as shown in a previous study [5]) may be related to other issues in addition to lack of knowledge, such as forgetting to report, lack of time, complexity or low user friendliness, lack of feedback on notified cases; some of these have already been reported in other countries for mandatory declaration diseases in general [54]. Even though CL is a non-notifiable disease in Portugal, it was considered to be otherwise by most physicians.

Perceptions of health professionals in terms of trend in number of cases in their region of work are compatible with the decrease in the number of VL cases reported annually in the period of 2014–2018 [4] compared to pre-2010 [5] and with increasing national canine seroprevalence [7, 8].

Inclusion of leishmaniasis in the curriculum and training of physicians and veterinarians is perceived as important, but for participants who recognize the disease as zoonotic, the lower rating for human health students and professionals could be related to the low incidence of the human disease [3] and to the fact that these cases are usually seen only by certain medical specialists. Although regional European guidelines for the management of leishmaniasis have been developed [1], most clinicians considered the creation of national guidelines to be very important and were not satisfied with the information available in official platforms.

Although systematic surveillance of phlebotomine sand flies is included in the Vector Surveillance Network (REVIVE [Rede de Vigilância de Vetores]), no national program is currently implemented to control leishmaniasis, which could explain why absence/inefficacy of vector control programs was often perceived as a barrier to controlling leishmaniasis.

Low adherence to protection measures in animals and insufficient vaccination coverage were also barriers often pointed by the veterinarians. In Portugal, although > 90% of dog owners seem to use ectoparasiticides for their dogs, the type of drug used and the frequency of application are often inappropriate to adequately prevent sand fly bites (15, 16). The use of vaccination as a preventative strategy is estimated at around 15% [7]. All of the interventions considered to be most effective (vaccination of animals, early diagnosis and treatment of diseased animals and use of repellents in animal pets) have been related in previous studies to control of the disease in diverse settings [55], except for the elimination of vector breeding sites.

Practices among physicians highlight that CL was less commonly seen, and that when seen it was often by professionals who also reported seeing cases of VL; it is possible that most diagnosed CL cases present simultaneous visceral involvement, as shown in data from inpatients in Portugal, where only 3/21 had isolated CL (5). It would appear likely that many cases of isolated, uncomplicated CL do not come to medical attention and are not diagnosed; it should be noted, however, that only one dermatologist was enrolled in the present study.

Diagnosis of VL has often relied on testing samples of bone marrow, although European guidelines suggest serology as the first-line approach [1], possibly because bone marrow aspirate or biopsy could be more informative in terms of differential diagnoses and also because serology may not be widely available. It should be noted that PCR was reported as the most used technique, which contrasts with data from 1999 to 2009 (use of PCR in only approx. 25% of cases [5]) and probably relates to a wider availability of this technique in more recent years. These assumptions are supported by the finding that availability was the factor most often selected as influencing the choice of diagnostic method. Preferential use of LAmB is consistent with previously reported data [5], and the seldom use of other regimens could also be related to weaker evidence for the use of miltefosine in the European region [1] and limited availability. Answers provided by physicians regarding CL suggest that each professional has seen few cases, reflecting low experience in diagnosing and treating this disease. Samples were most often reported to be obtained by excisional biopsy and tested by histopathology. A significant proportion of cases observed were suspected to originate from Latin America, consistent with an increasing migrant population from Brazil and more intense travel to the region [56]. Systematic identification of *Leishmania* species was reported by a minority of clinicians, suggesting treatment selection could be performed by inferring species identification based on geographical location. Additionally, monitoring of potential import and establishment of new species in the country is limited.

Concerning CanL, the use of serology for diagnosis was commonly reported, following the LeishVet diagnostic approach [52]. Treatment strategies favored allopurinol alone or allopurinol + MA, reflecting that veterinarians are likely following recommendations for mild or moderate/severe disease, respectively. It is interesting to note that 8/57 veterinarians reported having seen cases of feline leishmaniasis, reflecting a raising awareness of this endemic infection in cats and suggesting clinical cases are likely to be more common than reported [57].

The results of the present study overlap with findings from a previous Portuguese study that involved 141 veterinarians, with the results showing that > 50% of the veterinarians saw > 10 cases of leishmaniasis per year; serology was reported to be the preferred method for diagnosis (especially the immunofluorescence antibody test [IFAT]), followed by PCR in lymph nodes and/or bone marrow; allopurinol + MA was the most commonly used treatment regime. Two other recent national studies [21, 58] showed similar findings regarding diagnosis and treatment; additionally, in these latter studies, 31.3% of veterinarians reported not following any guidelines, even though 93.0% of responders were aware of their existence. In would appear that owner financial restraints negatively influenced veterinary follow-up and relapse recognition. Accordingly, two international studies including veterinarians in Portugal [59, 60] showed that veterinarians in Portugal took a relatively higher consideration of the impact of the socioeconomic situation on the veterinary care of dogs affected by leishmaniasis, in comparison to other countries. Additionally, these studies showed that rapid diagnostic testing was more commonly used for diagnosis in Portugal as compared to other European countries.

Globally, the approach to diagnosis and treatment of VL and CanL in Portugal generally follows regional (European) guidelines, but limitations in terms of access and lack of national specific recommendations could lead to some heterogeneity.

Median K and Per scores were significantly higher in professionals, and especially in animal and environmental health professionals, but this did not translate into higher Pra scores. A possible explanation is that the practices evaluated can be performed generally to prevent arthropod-borne infections, and not specifically for leishmaniasis (except for vaccination). It has been suggested in Brazil that veterinarians need to have increased knowledge on leishmaniases [30]. Results from the multivariate analysis reinforce the importance of practical experience as the means to increase knowledge in professionals and to promote that knowledge being effectively increased according to progression in disease course. Higher knowledge was associated with higher perception of the importance of education/training and of collaboration. Lower median Pra score in the Norte region could be related to a lower perception of risk for vector-borne infections, also likely associated with lower canine *Leishmania* seroprevalence in many subregions, especially in coastal areas [7].

This study has a number of important limitations. First, not every faculty in Portugal participated in the study. The courses that were contacted but did not collaborate, such as medial courses from Algarve, Madeira and Açores, were not actively contacted for collaboration in the study since they were not listed in the DGES website, which was used as a reference for this study [39]. In addition, adherence was generally very low. The participants of this questionnaire were probably students/professionals who were most likely to know/be aware of leishmaniasis (as the participation was voluntary, those who did not know/care about leishmaniasis might have been less likely to respond). Physicians and veterinarians were approached via social network publications due to visibility and adherence limitations with dissemination by email, and this could have resulted in a selection bias; professionals who take part in these Facebook® groups could be non-representative of the class in terms of age and other sociodemographic factors. Very few Internal Medicine physicians participated, although they likely represent the most involved specialty in diagnosis of adult VL in smaller hospitals. Similarly, the first approach to many patients, in primary health care, was not assessed since few family medicine physicians participated. Also, other professionals involved in healthcare were not enrolled in this study, such as nurses (human and veterinary), who are increasingly responsible for health education, but not involved in diagnosis/management or entomologic surveillance programs in Portugal.

The results of this study could also have been affected by social desirability or conformity bias, since some questions addressed points that could represent professional competence or student performance. Participation in the study was unsupervised, so individuals could have shared opinions or experiences or used external sources of information while filling in the questionnaire. Additionally, other biases associated with online participation could be presumed, including non-response bias and question order bias.

## Conclusions

Inclusion of leishmaniasis in the curriculum of health students is perceived as important and seems to be associated with an increase in knowledge of the disease. A national structured program to control leishmaniasis could overcome some of the barriers pointed out by professionals, namely by implementing systematic phlebotomine surveillance and integrated reporting of animal and human cases of disease. Joint efforts and collaboration are recognized as crucial to fight this zoonosis, following a One Health approach.

## Supplementary Information

**Additional file 1: Figure S1.** Online questionnaire about sociodemographic and professional/academic aspects and about knowledge, perceptions and practices regarding leishmaniasis

**Additional file 2: Table S1.** Protocol implemented for scoring knowledge, perceptions and practices of students and professionals, according to the answers provided in the questionnaire.

**Additional file 3: Figure S2.** Distribution of students by university and faculty of study for: **a** Integrated Master’s in Medicine, **b** Integrated Master’s in Veterinary Medicine, **c** Bachelor’s in Environmental Health

**Additional file 4: Figure S3.** Distribution of individual: **a** knowledge scores, **b** perceptions scores, **c** practices scores

## Data Availability

The datasets generated and analyzed during the current study are not publicly available due to confidentiality commitment with the participants, as stated in the consent declaration, but are available from the corresponding author on reasonable request.
