# Supplementary material for: Knowledge, perceptions and practices of health students and professionals regarding leishmaniasis in Portugal: a cross-sectional study
Source: Parasit Vectors. 2023 Oct 25;16:381. doi: 10.1186/s13071-023-05982-z (PMC10598964; doi:10.1186/s13071-023-05982-z)
Supplement: Supplementary file 1 — Additional file 1: Figure S1. Online questionnaire about sociodemographic and professional/academic aspects and about knowledge, perceptions and practices regarding leishmaniasis [file 13071_2023_5982_MOESM1_ESM.docx]

**Supplementary figure 1**

Online questionnaire about sociodemographic and professional/academic aspects and about knowledge, perceptions and practices regarding leishmaniasis

The original questionnaire (in Portuguese) can be accessed using the following link: https://redcap.ihmt.unl.pt/redcap/surveys/?s=EDR3NKAHCK

As an example, a translation into English of the following elements of the questionnaire is presented below:

1. Information to participant and consent form
2. Complete set of questions potentially presented to Veterinary Medicine students
3. Complete set of questions potentially presented to Veterinarians

**1)**

**Knowledge, perceptions and practices of health students and professionals regarding leishmaniasis in Portugal**

We request your participation in a study. Please read the following information carefully. The aim of this study is to investigate the knowledge, perceptions and practices of health students and professionals (human, animal and environmental) in Portugal regarding leishmaniasis. The following questionnaire was designed for this purpose and was sent to medical doctors, veterinarians, environmental health technicians and students in each of these areas, in different regions of the country. By completing this questionnaire, you will be contributing to the development of means to strengthen the knowledge of leishmaniasis among future health professionals and to improve the diagnostic and therapeutic guidance, as well as investing in forms of prevention provided by health care. This study is coordinated by Rafael Rocha (leish.rar@ihmt.unl.pt), physician at Centro Hospitalar Universitário de São João, Porto, and PhD student at the Instituto de Higiene e Medicina Tropical (IHMT), Universidade NOVA de Lisboa, and by the professors and researchers at IHMT NOVA: Carla Maia (carlamaia@ihmt.unl.pt), Cláudia Conceição (claudiaconceicao@ihmt.unl.pt), Luzia Gonçalves ([luziag@ihmt.unl.pt](mailto:luziag@ihmt.unl.pt)). In case of any questions or concerns regarding the study, please contact us through the emails provided, preferably with Carla Maia. This study was authorized by the Scientific Council of the IHMT and received a favorable opinion from the Ethics Committee of the IHMT. The estimated time for completing this questionnaire is 7-15 minutes. Participation is completely voluntary, with no expected risks and no immediate benefit. You can interrupt your participation at any time by closing your browser window. In that case, your responses will not be recorded. After clicking on the “Submit” button at the end of the questionnaire, it will no longer be possible to withdraw your information from the study, due to the impossibility of identifying your answer. The collected data will be preserved for a maximum period of 5 years after the publication of the study. The information you will provide will be anonymous. When submitting the form, the email used will not be recorded. The following questions do not allow for personal identification. The submitted answers will be stored online, on a platform with access restricted by password, only accessible to the researchers of this study. The processing of the information carried out will serve exclusively for scientific purposes. If you wish to receive the results of this study, given that the study is anonymous, please contact the team using the emails provided. By starting to fill in this questionnaire, you indicate that you have read and understood the above information and you agree to read all the information carefully and answer all the questions carefully and honestly.

I consider myself duly informed about the objectives of the study and what is asked of me. I was guaranteed the confidentiality of data and the voluntary participation and the possibility of withdrawing from participating:

- Yes
- No

Please select the category to which you belong:

- Medical doctor
- Veterinary doctor
- Environmental health technician
- Medicine student
- Veterinary medicine student
- Environmental health student
- None of the above

**2)**

**Sociodemographic data**

**Are you currently enrolled in an Integrated Master’s in Veterinary Medicine in Portugal?**

- Yes
- No

**Age (years): ____**

**Gender:**

- Male
- Female
- Other

**Current district of residence: ____**

**Institution in which you are enrolled in the Integrated Master’s in Veterinary Medicine:**

- Universidade do Porto - Instituto de Ciências Biomédicas Abel Salazar
- Universidade de Lisboa - Faculdade de Medicina Veterinária
- Universidade de Trás-os-Montes e Alto Douro - Escola de Ciências Agrárias e Veterinárias
- Universidade de Évora - Escola de Ciências e Tecnologia
- Escola Universitária Vasco da Gama
- Instituto Universitário Egas Moniz
- Instituto Universitário de Ciências da Saúde
- Universidade Lusófona de Humanidades e Tecnologias
- Other

**Degree of the course you are currently enrolled in:**

- 1^st^
- 2^nd^
- 3^rd^
- 4^th^
- 5^th^
- 6^th^

**Knowledge, perceptions and practices regarding leishmaniasis**

(Questions marked with * allow selection of multiple answers)

**Have you ever heard of leishmaniasis?**

- Yes, in humans and animals
- Yes, in animals
- Yes, in humans
- No
- Don’t know/Can’t remember

**Have you ever heard of animal leishmaniasis during the Integrated Master’s in Veterinary Medicine?**

- Yes
- No
- Don’t know/Can’t remember

***Select the context(s) during the Integrated Master’s in Veterinary Medicine in which you have heard about animal leishmaniasis:**

- Informal talk with professors
- Informal talk with colleagues
- Theoretical classes
- Practical classes – leishmaniasis laboratory diagnostic methods
- Practical classes – contact with animals/tutors
- Practical classes – clinical cases about leishmaniasis
- Research project or presentation for a subject
- Courses/workshops/congresses where leishmaniasis was addressed
- Other
- Don’t know/Can’t remember

***Select the year(s) of the Integrated Master’s in Veterinary Medicine in which you have heard about leishmaniasis:**

- 1^st^
- 2^nd^
- 3^rd^
- 4^th^
- 5^th^
- 6^th^
- Don’t know/Can’t remember

**Have you ever heard of animal leishmaniasis outside the academic context?**

- Yes
- No
- Don’t know/Can’t remember

***Select the situations in which you have heard about animal leishmaniasis, outside the academic context:**

- I have/had a domestic animal diagnosed with leishmaniasis
- Television advertisements
- Poster advertisements
- News on television or journal
- Conversation with friends
- Conversation with family
- Conversation with a veterinary doctor
- Search on the internet
- Through social networks
- Other
- Don’t know/Can’t remember

**What type of agent causes leishmaniasis?**

- Virus
- Bacterium
- Parasite
- Fungus
- Don’t know/Can’t remember

**Can the species causing leishmaniasis in animals also cause disease in humans?**

- Yes
- No, they are different species
- Don’t know/Can’t remember

***Which of the following is/are frequent route(s) of transmission of leishmaniasis?**

- Mosquito bite
- Sand fly bite
- Flea bite
- Tick bite
- Direct contact with animals and/or their secretions
- Animal bite/scratch
- Consumption of contaminated water/food
- Sexual contact
- Vertical (mother-child)
- Organ transplant
- Blood transfusion
- Don’t know/Can’t remember

**Is animal leishmaniasis diagnosed in Portugal?**

- Yes, and the infection can be acquired in Portugal
- Yes, but the infection is always acquired outside Portugal
- No, Don’t know/Can’t remember

***Which of these do you consider to be the animal(s) most affected by leishmaniasis in Portugal (in terms of number of cases diagnosed per year)?**

- Dogs
- Cats
- Horses
- Livestock (bovine, ovine, caprine)
- Rabbits/hares
- Wild carnivores
- Other wild mammals
- Others
- Don’t know/Can’t remember

**What do you consider to be the risk of an animal developing leishmaniasis in the region where you study?**

- None
- Low
- Medium
- High
- Don’t know/Can’t remember

***Which of the following environmental/behavioral factors do you associate with a higher risk of transmission of leishmaniasis?**

- Living in a rural environment
- Living in an animal shelter
- Hunting, shepherd or guard activities
- Staying mostly outdoors
- Sharing a home with an animal with leishmaniasis
- Consuming non-packaged food items
- Non-systematic use of repellents
- Living in a home/environment with accumulation of organic matter
- Living in an environment close to water
- Don’t know/Can’t remember

***Which of the following animal factors do you consider to confer a higher risk for the development of leishmaniasis?**

- Immunosuppressive therapy
- Breed
- Male sex
- Old age
- Young/juvenile
- Don’t know/Can’t remember

**Have you ever been involved in the diagnosis and treatment of any animal with leishmaniasis, during your academic path?**

- Yes
- No
- Don’t know/Can’t remember

**What is the estimate of the number of animals with leishmaniasis in which you were involved in the diagnosis and/or treatment?**

- 1
- 2-5
- 6-10
- 11-20
- 21-50
- >50
- Don’t know/Can’t remember

***Which sign(s) do you consider to be more frequent in animal leishmaniasis?**

- Skin changes
- Mucosal lesions
- Ocular changes
- Nail changes (for example, onychogryphosis)
- Weight loss
- Fever
- Weakness/fatigue
- Muscular atrophy
- Vomiting
- Diarrhea
- Nasal hemorrhage (epistaxis)
- Increased lymph node size (lymphadenopathy)
- Increased spleen size (splenomegaly)
- Increased liver size (hepatomegaly)
- Other
- Don’t know/Can’t remember

**The infection by agents causing leishmaniasis in animals is:**

- Always symptomatic
- Mostly symptomatic
- Mostly asymptomatic
- Don’t know/Can’t remember

**Which of the following laboratory exams do you think is most frequently used to diagnose leishmaniasis in animals?**

- Culture
- Microscopy of smear/histology
- DNA detection (by PCR)
- Serology
- Don’t know/Can’t remember

***Which of the following types of samples do you think is/are most frequently used to diagnose leishmaniasis in animals?**

- Blood
- Bone marrow biopsy/aspirate
- Skin biopsy/cytology
- Spleen biopsy/aspirate
- Lymph node biopsy/aspirate
- Don’t know/Can’t remember

**Is there any treatment for leishmaniasis in animals?**

- Yes
- No
- Don’t know/Can’t remember

**What is your perception of the global effectiveness of anti-leishmaniasis treatments?**

**On the resolution of signs/clinical improvement:**

- Not effective
- Slightly effective
- Moderately effective
- Very effective
- Extremely effective
- Don’t know/Can’t remember

**On the cure of infection**

- Not effective
- Slightly effective
- Moderately effective
- Very effective
- Extremely effective
- Don’t know/Can’t remember

**Is there any vaccine against leishmaniasis in dogs, in Portugal?**

- Yes
- No
- Don’t know/Can’t remember

***In which context(s) have you heard of human leishmaniasis?**

- In the Veterinary Medicine degree
- In another degree I was previously enrolled in
- I know a person who has/had leishmaniasis
- Television advertisements
- Poster advertisements
- News on television or journal
- Conversation with friends
- Conversation with family
- Conversation with a medical doctor
- Search on the internet
- Through social networks
- Other
- Don’t know/Can’t remember

**Is human leishmaniasis diagnosed in Portugal?**

- Yes
- No
- Don’t know/Can’t remember

**Is human leishmaniasis curable?**

- No, and there is currently no treatment
- No, but there is chronic treatment
- Yes
- Don’t know/Can’t remember

***Which of the following measures do you think could play a more relevant role in the control of leishmaniasis?**

- Use of mosquito nets in animal shelters
- Use of repellents/insecticides in pet animals
- Use of environmental repellents/insecticides (in closed spaces)
- Vaccination of animals against leishmaniasis
- Elimination of vector breeding sites
- Early diagnosis and treatment of cases of animal leishmaniasis
- Culling of animals diagnosed with leishmaniasis
- Avoiding travelling to highly endemic areas
- Don’t know/Can’t remember

**How important do you think is the inclusion of leishmaniasis in the curriculum program of:**

**Veterinary Medicine**

- Not important
- Slightly important
- Moderately important
- Very important
- Extremely important
- Don’t know

**Medicine**

- Not important
- Slightly important
- Moderately important
- Very important
- Extremely important
- Don’t know

**What is your degree of satisfaction with the quantity and quality of information about leishmaniasis provided, until now, in the Master’s Degree of Veterinary Medicine?**

- Not satisfied
- Slightly satisfied
- Moderately satisfied
- Very satisfied
- That subject has never been addressed
- Don’t know

**How important do you think is the collaboration between doctors, veterinarians and biologists/ecologists to eliminate leishmaniasis?**

- Not important
- Slightly important
- Moderately important
- Extremely important
- Don’t know/No opinion

**Do you use insect repellent/insecticide when you perform outdoor activities during the night?**

- Yes, always
- Yes, sometimes
- No
- I rarely/never perform activities outdoors during the night
- Don’t know/Can’t remember

**Does your home have nets on the windows and/or doors?**

- Yes, in all
- Yes, in some
- No
- Don’t know/Can’t remember

**Do you have pet animals?**

- Yes – dog(s)
- Yes – other(s)
- No
- Don’t know/Can’t remember

***Do(es) your dog(s) spend time outdoors between dusk and dawn?**

- Yes – in the garden/backyard
- Yes – on the street
- Yes – in a forest/bush area
- Yes – other
- No
- Don’t know/Can’t remember

**Do(es) your dog(s) use insect repellent/insecticide products?**

- Yes, all year round
- Yes, in some months of the year
- No
- Don’t know/Can’t remember

***Select the product(s) your dog(s) use(s) most frequently:**

- Collar
- Spot-on
- Spray
- Shampoo
- Pills
- Other(s)
- Don’t know/Can’t remember

**Is/are your dog(s) vaccinated against leishmaniasis?**

- Yes – every year
- Yes – some years
- No
- Don’t know/Can’t remember

**Is/are your dog(s) regularly seen by a veterinarian?**

- Yes – more than once per year
- Yes – approximately once per year
- Yes – every 2 years
- No
- Don’t know/Can’t remember

**You have finished filling in this questionnaire.**

Please, click on the “Submit” button to submit your answers. You will be directed to a final acknowledgment page, where you will find the contacts of the organizing team of this study. These contacts are also visible in the email you received with the disclosure of this study.

**3)**

**Sociodemographic data**

(Questions marked with * allow selection of multiple answers)

**Are you currently practicing Veterinary Medicine in Portugal?**

- Yes
- No

**Age (years): ____**

**Gender:**

- Male
- Female
- Other

**Current district of residence: ____**

**Type of veterinary unit where you mostly work on:**

- Veterinary care center
- Municipal veterinarian
- Zoo or similar
- Wildlife recovery center
- Other

**Current district of workplace: ____**

***What group(s) of animals do you predominantly work with?**

- Companion animals (dogs and cats)
- Horses
- Livestock (cattle, goats, sheep)
- Exotics
- Other
- Not applicable

***Type of service you work in (please select all that apply):**

- Emergency Service
- Inpatient ward
- Consultations
- Laboratory
- Imaging diagnostic methods
- Other

**Professional experience:**

- < 5 years
- 5 to 9 years
- 10 to 14 years
- 15 to 19 years old
- 20 or more years

**Institution where you attended the Veterinary Medicine course:**

- Universidade do Porto - Instituto de Ciências Biomédicas Abel Salazar
- Universidade de Lisboa - Faculdade de Medicina Veterinária
- Universidade de Trás-os-Montes e Alto Douro - Escola de Ciências Agrárias e Veterinárias
- Universidade de Évora - Escola de Ciências e Tecnologia
- Escola Universitária Vasco da Gama
- Instituto Universitário Egas Moniz
- Instituto Universitário de Ciências da Saúde
- Universidade Lusófona de Humanidades e Tecnologias
- Other

**Knowledge, perceptions and practices regarding leishmaniasis**

(Questions marked with * allow selection of multiple answers)

**Have you ever heard of leishmaniasis?**

- Yes, in humans and animals
- Yes, in animals
- Yes, in humans
- No
- Don’t know/Can’t remember

**Have you ever diagnosed animals with leishmaniasis in Portugal in your professional activity?**

- Yes
- No
- Don’t know/Can’t remember

***In which context(s) have you heard of animal leishmaniasis?**

- In the Veterinary Medicine course
- In my professional activity
- Outside the academic/professional context (example, social interaction)
- Don’t know/Can’t remember

***Select the context(s) in which you have heard about animal leishmaniasis, in your professional activity:**

- Contact/observation of animals with leishmaniasis
- Case notification/declaration
- Integration in research team
- Performance of a diagnostic test
- Courses/trainings/conferences that addressed the disease
- Conversation with colleagues
- Conversation with pet tutors
- Other
- Don’t know/Can’t remember

***Select the situations in which you have heard about animal leishmaniasis, outside the academic/professional context:**

- I have/had a domestic animal diagnosed with leishmaniasis (by another professional)
- Television advertisements
- Poster advertisements
- News on television or journal
- Conversation with friends
- Conversation with family
- Search on the internet
- Through social networks
- Other
- Don’t know/Can’t remember

**Is animal leishmaniasis diagnosed in Portugal?**

- Yes, and the infection can be acquired in Portugal
- Yes, but the infection is always acquired outside Portugal
- No, Don’t know/Can’t remember

***In which context(s) have you heard of human leishmaniasis?**

- I know a person who has/had leishmaniasis
- In my professional activity
- Television advertisements
- Poster advertisements
- News on television or journal
- Conversation with friends
- Conversation with family
- Conversation with a medical doctor
- Search on the internet
- Through social networks
- Other
- Don’t know/Can’t remember

**Is human leishmaniasis diagnosed in Portugal?**

- Yes, and the infection can be acquired in Portugal
- Yes, but the infection is always acquired outside Portugal
- No, Don’t know/Can’t remember

**What type of agent causes leishmaniasis?**

- Virus
- Bacterium
- Protozoan parasite
- Helminth parasite
- Fungus
- Don’t know/Can’t remember

**How many species of pathogenic agent of leishmaniasis can cause disease in animals?**

- Only one
- Several, but in Europe cases are caused mostly by 1 species
- Several and in Europe cases are caused by several species
- Don’t know/Can’t remember

**Can the species causing leishmaniasis in animals also cause disease in humans?**

- Yes
- No, they are different species
- Don’t know/Can’t remember

***Which of the following is/are frequent route(s) of transmission of leishmaniasis?**

- Mosquito bite
- Sand fly bite
- Flea bite
- Tick bite
- Direct contact with animals and/or their secretions
- Animal bite/scratch
- Consumption of contaminated water/food
- Sexual contact
- Vertical (mother-child)
- Organ transplant
- Blood transfusion
- Don’t know/Can’t remember

**What do you think is the origin of the cases of animal leishmaniasis diagnosed in Portugal?**

- All autochthonous
- Mostly autochthonous
- Equal proportion of autochthonous and imported
- Mostly imported
- All imported
- Don’t know/Can’t remember

***Which of these do you consider to be the animal(s) most affected by leishmaniasis in Portugal (in terms of number of cases diagnosed per year)?**

- Dogs
- Cats
- Horses
- Livestock (bovine, ovine, caprine)
- Rabbits/hares
- Wild carnivores
- Other wild mammals
- Others
- Don’t know/Can’t remember

**How many cases of leishmaniasis in animals do you think are diagnosed, per year, in the district where you work?**

- 1-10
- 11-20
- 21-50
- 51-100
- >100
- Don’t know/Can’t remember

**What is your perception regarding the evolution in the number of cases diagnosed in animals, in the district where you work, in the last 10 years?**

- Increase in cases
- Reduction in cases
- No increase nor reduction of cases
- Don’t know/No opinion

***Which of the following environmental/behavioral factors do you associate with a higher risk of transmission of leishmaniasis in dogs?**

- Living in a rural environment
- Living in an animal shelter
- Hunting, shepherd or guard activities
- Staying mostly outdoors
- Sharing a home with an animal with leishmaniasis
- Sharing a home with a person with leishmaniasis
- Consuming non-packaged food items
- Non-systematic use of repellents
- Living in a home/environment with accumulation of organic matter
- Living in an environment close to water
- Don’t know/Can’t remember

**What do you consider to be the importance of the following host (dog) factors for the development of leishmaniasis?**

|  | None | Little | Medium | Great | Extreme |
| --- | --- | --- | --- | --- | --- |
| Immunosuppressive therapy |  |  |  |  |  |
| Diabetes mellitus |  |  |  |  |  |
| Breed |  |  |  |  |  |
| Male sex |  |  |  |  |  |
| Advanced age |  |  |  |  |  |
| Young/juvenile |  |  |  |  |  |

**Which sign(s) and analytical changes do you consider to be more frequent in leishmaniasis in dogs?**

|  | Never | Rare | Infrequent | Moderately frequent | Very frequent | Always | Don’t know  Can’t remember |
| --- | --- | --- | --- | --- | --- | --- | --- |
| Fever |  |  |  |  |  |  |  |
| Weakness/fatigue |  |  |  |  |  |  |  |
| Weight loss |  |  |  |  |  |  |  |
| Skin manifestations |  |  |  |  |  |  |  |
| Mucosal lesions |  |  |  |  |  |  |  |
| Ocular changes |  |  |  |  |  |  |  |
| Onychogryphosis |  |  |  |  |  |  |  |
| Vomiting/diarrhea |  |  |  |  |  |  |  |
| Epistaxis |  |  |  |  |  |  |  |
| Lymphadenopathy |  |  |  |  |  |  |  |
| Hepato and/or splenomegaly |  |  |  |  |  |  |  |
| Pancytopenia |  |  |  |  |  |  |  |
| Renal dysfunction |  |  |  |  |  |  |  |
| Hypergammaglobulinemia |  |  |  |  |  |  |  |

**The infection by agents causing leishmaniasis in animals is:**

- Always symptomatic
- Mostly symptomatic
- Mostly asymptomatic
- Don’t know/Can’t remember

**How often do you use the following types of samples for diagnosing leishmaniasis in dogs?**

|  | Never | Sometimes | Often | Always | Don’t know  Can’t remember |
| --- | --- | --- | --- | --- | --- |
| Bone marrow biopsy/aspirate |  |  |  |  |  |
| Lymph node biopsy/aspirate |  |  |  |  |  |
| Biopsy/cytology of skin |  |  |  |  |  |
| Spleen biopsy/aspirate |  |  |  |  |  |
| Blood |  |  |  |  |  |

**How often do you ask/recommend the following laboratory exams for diagnosing leishmaniasis in dogs? (**PCR - Polymerase Chain Reaction; ELISA - Enzyme-Linked Immunosorbent Assay; IFAT - Immunofluorescence Antibody Test)

|  | Never | Sometimes | Often | Always | Don’t know  Can’t remember |
| --- | --- | --- | --- | --- | --- |
| Culture exam |  |  |  |  |  |
| Microscopic exam of smear/histology |  |  |  |  |  |
| PCR |  |  |  |  |  |
| Quantitative serology (e.g. ELISA/IFAT) |  |  |  |  |  |
| Qualitative serology (rapid test) |  |  |  |  |  |

**Is notification of cases of leishmaniasis diagnosed in animals mandatory in Portugal?**

- Sim
- No
- Don’t know/Can’t remember

**Do you recommend any treatment, for the animals you diagnose with *Leishmania* infection?**

- Sim – always
- Sim – if they are symptomatic (severe)
- Sim – if they are symptomatic (mild or severe)
- No
- Don’t know/Can’t remember

**When treating dogs with leishmaniasis, how often have you used the following drugs or drug combinations?**

|  | Never | In some cases | About half of the cases | In most cases | In all cases | Don’t know  Can’t remember |
| --- | --- | --- | --- | --- | --- | --- |
| Allopurinol |  |  |  |  |  |  |
| Meglumine antimoniate |  |  |  |  |  |  |
| Miltefosine |  |  |  |  |  |  |
| Allopurinol+meglumine antimoniate |  |  |  |  |  |  |
| Allopurinol+miltefosine |  |  |  |  |  |  |
| Other |  |  |  |  |  |  |

**If you answered "Other" in the previous question, please specify: ____**

**What is your perception of the effectiveness of anti-leishmaniasis treatments? (select the option "not applicable" if you do not use the indicated treatments)**

|  | Not effective | Slightly effective | Moderately effective | Very effective | Extremely effective | Not applicable  Don’t know |
| --- | --- | --- | --- | --- | --- | --- |
| Allopurinol in mild/moderate disease |  |  |  |  |  |  |
| Allopurinol in severe/very severe disease |  |  |  |  |  |  |
| Miltefosine in mild/moderate disease |  |  |  |  |  |  |
| Miltefosine in severe/very severe disease |  |  |  |  |  |  |
| Antimoniate in mild/moderate disease |  |  |  |  |  |  |
| Antimoniate in severe/very severe disease |  |  |  |  |  |  |
| Allopurinol+antimoniate in mild/moderate disease |  |  |  |  |  |  |
| Allopurinol+antimoniate in severe/very severe disease |  |  |  |  |  |  |
| Miltefosine+antimoniate in mild/moderate disease |  |  |  |  |  |  |
| Miltefosine+antimoniate in severe/very severe disease |  |  |  |  |  |  |

**Is there any vaccine against leishmaniasis in dogs, in Portugal?**

- Yes
- No
- Don’t know/Can’t remember

**Do you recommend vaccination against leishmaniasis in dogs, in the area where you work?**

- Yes – for every dog
- Yes – for some dogs
- No
- Don’t know/Not applicable

**How effective do you think the following measures are for controlling leishmaniasis?**

|  | Not effective | Slightly effective | Moderately effective | Very effective | Extremely effective | Don’t know  Can’t remember |
| --- | --- | --- | --- | --- | --- | --- |
| Use of repellents/insecticides in pet animals |  |  |  |  |  |  |
| Use of environmental repellents/insecticides (in closed spaces) |  |  |  |  |  |  |
| Use of immunoprophylactic scheme with domperidone in dogs in endemic areas |  |  |  |  |  |  |
| Vaccination of dogs against leishmaniasis |  |  |  |  |  |  |
| Elimination of vector breeding sites |  |  |  |  |  |  |
| Early diagnosis and treatment of symptomatic cases of animal leishmaniasis |  |  |  |  |  |  |
| Screening and treatment of asymptomatic infected dogs with risk factors for progression (immunosuppressed, for example) |  |  |  |  |  |  |
| Culling of animals diagnosed with leishmaniasis |  |  |  |  |  |  |
| Avoiding travelling to highly endemic areas |  |  |  |  |  |  |

**In your clinical practice, how often do you recommend to tutors individual measures to prevent leishmaniasis?**

- Never
- Sometimes
- Often
- Always
- Don't know/Can't remember

**How many cases of leishmaniasis in dogs have you diagnosed in your professional activity?**

- 1
- 2-5
- 6-10
- 11-20
- 21-50
- >50
- Don't know/Can't remember

**Have you ever diagnosed leishmaniasis in other animal species? If yes, please specify which species and number of cases: ____**

**How important do you think is the inclusion of leishmaniasis in the curriculum program of:**

|  | Not important | Slightly important | Moderately important | Very important | Extremely important | Don’t know |
| --- | --- | --- | --- | --- | --- | --- |
| Veterinary Medicine students |  |  |  |  |  |  |
| Veterinarians, in the workplace |  |  |  |  |  |  |
| Medicine students |  |  |  |  |  |  |

**Do you consider that there is enough information on animal leishmaniasis available on official platforms?**

- Yes
- No
- Don't know/Can't remember

**How important do you think is the collaboration between doctors, veterinarians and biologists/ecologists to eliminate leishmaniasis?**

- Not important
- Slightly important
- Moderately important
- Extremely important
- Don’t know/No opinion

**How important do you think would be the existence of a structured national program, bringing together human and animal health entities, for the elimination of leishmaniasis in Portugal?**

- Not important
- Slightly important
- Moderately important
- Extremely important
- Don’t know/No opinion

***What do you consider to be the main barrier(s) in the fight against leishmaniasis in Portugal?**

- Lack of knowledge of the disease by the general population
- Lack of knowledge of the disease by animal health professionals
- Lack of knowledge of the disease by human health professionals
- Failures in the early diagnosis and treatment of cases of leishmaniasis in domestic animals
- Failures in the control of leishmaniasis in wild animals
- Ineffectiveness/Absence of vector control programs
- Poor adherence to measures to protect domestic animals (repellents)
- Poor adherence to individual protection measures in the human population at risk
- Insufficient vaccination coverage of domestic animals at risk
- Increasing number of animals and people with risk factors for leishmaniasis
- Risk of importing the disease through travel and migration
- Unavailability of diagnostic tools in hospitals and veterinary clinics
- Unavailability of more forms of treatment in Portugal
- Insufficient research in the field of leishmaniasis
- Absence of a systematic reporting system for cases of leishmaniasis in animals
- Don't know/Can't remember

**Do you use insect repellent/insecticide when you perform outdoor activities during the night?**

- Yes, always
- Yes, sometimes
- No
- I rarely/never perform activities outdoors during the night
- Don’t know/Can’t remember

**Does your home have nets on the windows and/or doors?**

- Yes, in all
- Yes, in some
- No
- Don’t know/Can’t remember

**Do you have pet animals?**

- Yes – dog(s)
- Yes – other(s)
- No
- Don’t know/Can’t remember

***Do(es) your dog(s) spend time outdoors between dusk and dawn?**

- Yes – in the garden/backyard
- Yes – on the street
- Yes – in a forest/bush area
- Yes – other
- No
- Don’t know/Can’t remember

**Do(es) your dog(s) use insect repellent/insecticide products?**

- Yes, all year round
- Yes, in some months of the year
- No
- Don’t know/Can’t remember

***Select the product(s) your dog(s) use(s) most frequently:**

- Collar
- Spot-on
- Spray
- Shampoo
- Pills
- Other(s)
- Don’t know/Can’t remember

**Is/are your dog(s) vaccinated against leishmaniasis?**

- Yes – every year
- Yes – some years
- No
- Don’t know/Can’t remember

**Is/are your dog(s) regularly seen by a veterinarian?**

- Yes – more than once per year
- Yes – approximately once per year
- Yes – every 2 years
- No
- Don’t know/Can’t remember

**You have finished filling in this questionnaire.**

Please, click on the “Submit” button to submit your answers. You will be directed to a final acknowledgment page, where you will find the contacts of the organizing team of this study. These contacts are also visible in the email you received with the disclosure of this study.
