# Supplementary material for: Knowledge, perceptions and practices of health students and professionals regarding leishmaniasis in Portugal: a cross-sectional study
Source: Parasit Vectors. 2023 Oct 25;16:381. doi: 10.1186/s13071-023-05982-z (PMC10598964; doi:10.1186/s13071-023-05982-z)
Supplement: Supplementary file 2 — Additional file 2: Table S1. Protocol implemented for scoring knowledge, perceptions and practices of students and professionals, according to the answers provided in the questionnaire. [file 13071_2023_5982_MOESM2_ESM.docx]

**Supplementary table 1**

Protocol implemented for scoring Knowledge, Perceptions and Practices of students and professionals, according to the answers provided in the questionnaire

1. Knowledge score

Total knowledge score for each student/professional obtained by adding individual question scores

Higher scores representing higher level of knowledge

(Minimum 0 – Maximum 10)

| Question description | Answer(s) selected | Score |
| --- | --- | --- |
| Have you ever heard of leishmaniasis? | Yes - leishmaniasis in humans and animals | 2 |
|  | Yes – leishmaniasis in animals  Yes – leishmaniasis in humans | 1 |
|  | No  Don’t know/Can’t remember  No answer | 0 |
| What type of agent causes leishmaniasis? | Parasite  Protozoan parasite | 1 |
|  | Helminth parasite | 0.5 |
|  | Bacteria  Virus  Fungus  Yeast fungus  Mold fungus  Don’t know/Can’t remember  No answer | 0 |
| Which of the following is/are frequent route(s) of transmission of leishmaniasis? | Sand fly bite + 0 | 2 |
|  | Sand fly bite + Any | 1.5 |
|  | Mosquito bite ± Any except Sand fly bite | 1 |
|  | Flea bite or Tick bite ± Any except Sand fly bite or Mosquito bite | 0.5 |
|  | All other answers  No answer | 0 |
| Which of the following is/are frequent route(s) of transmission of leishmaniasis?^1^ | Insect/arthropod bite + 0 | 1 |
|  | Insect/arthropod bite + Any | 0.5 |
|  | Any except Insect/arthropod bite  No answer | 0 |
| What is the insect/arthropod vector of leishmaniasis?^1^ | Sandfly + 0 | 1 |
|  | Sandfly + Any  Mosquito + 0 | 0.5 |
|  | Mosquito + Any  Any except Sand fly or Mosquito  No answer | 0 |
| Is animal leishmaniasis diagnosed in Portugal? | Yes, and the infection can be acquired in Portugal  Yes | 1 |
|  | Yes, but the infection is always acquired abroad | 0.5 |
|  | No  Don’t know/Can’t remember  No answer | 0 |
| Is human leishmaniasis diagnosed in Portugal? | Yes, and the infection can be acquired in Portugal  Yes | 1 |
|  | Yes, but the infection is always acquired abroad | 0.5 |
|  | No  Don’t know/Can’t remember  No answer | 0 |
| Is leishmaniasis diagnosed in Portugal?^1,2^ | Yes, and the infection can be acquired in Portugal | 2 |
|  | Yes, but the infection is always acquired abroad | 1 |
|  | No  Don’t know/Can’t remember  No answer | 0 |
| Is visceral leishmaniasis diagnosed in Portugal?  +  Is cutaneous leishmaniasis diagnosed in Portugal? ^3,4^ | Selects “Yes, and all cases are acquired in Portugal/All autochthonous” in at least one of the questions  Selects “Yes, and most cases are acquired in Portugal/Most autochthonous” in at least one of the questions  Selects “Yes, same proportion autochthonous and imported” in at least one of the questions”  Selects “Yes, and most cases are imported/Most imported” in at least one of the questions | 1 |
|  | Yes, and all cases are imported/All imported + Yes, and all cases are imported/All imported  Selects “Yes+Don’t know/can’t remember” or “Yes+No answer” in both questions | 0.5 |
|  | Any combination of No, Don’t know/Can’t remember or No answer in the two questions | 0 |
| Which of these do you consider to be the animal(s) most affected by leishmaniasis in Portugal (in terms of number of cases diagnosed per year)? | Dog + 0 | 1 |
|  | Dog + Any | 0.5 |
|  | All other answers  No answer | 0 |
| The infection by agents causing leishmaniasis in animals/humans is: | Mostly asymptomatic | 1 |
|  | Mostly symptomatic | 0.5 |
|  | Always symptomatic  Don’t know/Can’t remember  No answer | 0 |
| Is there any treatment for leishmaniasis in animals? | Yes | 1 |
|  | No  Don’t know/Can’t remember  No answer | 0 |
| Is there any treatment for leishmaniasis in humans?  Or  Is there any drug treatment for visceral leishmaniasis? ^3^ | Yes | 1 |
|  | No  Don’t know/Can’t remember  No answer | 0 |
| Is visceral leishmaniasis curable? ^4,6^ | Yes  No, but there is chronic treatment | 1 |
|  | No, and there is currently no treatment  Don’t know/Can’t remember  No answer | 0 |
| Is there any treatment for leishmaniasis in dogs? ^5^ | Yes – there are several drugs available in Portugal  Yes – there is only 1 drug available in Portugal  Yes – but no drug is available in Portugal | 1 |
|  | No  Don’t know/Can’t remember  No answer | 0 |
| Have you ever been involved in the diagnosis and treatment of a patient with visceral/cutaneous/leishmaniasis? ^3^  Or  In your professional activity, have you ever diagnosed any animal with leishmaniasis, in Portugal? ^5^ | Yes | 1 |
|  | No  Don’t know/Can’t remember  No answer | 0 |

^1^ – Questions only presented in the Environmental Health technicians’ questionnaire

^2^ – Questions only presented in the Environmental Health students’ questionnaire

^3^ - Questions only presented in the medical doctors’ questionnaire

^4^ - Questions only presented in the Medicine students’ questionnaire

^5^ – Questions only presented in the veterinary doctors’ questionnaire

^6^ - Questions only presented in the Veterinary Medicine students’ questionnaire

1. Perceptions score

Total perceptions score for each student/professional obtained by adding individual question scores

Higher scores representing higher level of perception of importance of training and collaboration

(Minimum 0 – Maximum 11)

| Question description | Answer(s) selected | Score |
| --- | --- | --- |
| How important do you think is the inclusion of leishmaniasis in the curriculum program of Veterinary Medicine? | Extremely important | 4 |
|  | Very important | 3 |
|  | Moderately important | 2 |
|  | Slightly important | 1 |
|  | Not important  Don’t know  No answer | 0 |
| How important do you think is the inclusion of leishmaniasis in the curriculum program of Medicine? | Extremely important | 4 |
|  | Very important | 3 |
|  | Moderately important | 2 |
|  | Slightly important | 1 |
|  | Not important  Don’t know  No answer | 0 |
| How important do you think is the collaboration between doctors, veterinarians, and biologists/ecologists/environmental health technicians to eliminate leishmaniasis? | Extremely important | 3 |
|  | Moderately important | 2 |
|  | Slightly important | 1 |
|  | Not important  Don’t know/No opinion  No answer | 0 |

1. Practices score

Total practices score for each student/professional obtained by adding individual question scores

Higher scores representing higher level of protective practices

(Minimum 0 – Maximum 4)

| Question description | Answer(s) selected | Score |
| --- | --- | --- |
| Do you use insect repellent/insecticide when you perform outdoor activities during the night? | Yes, always  I rarely/never perform activities outdoors during the night | 1 |
|  | Yes, sometimes | 0.5 |
|  | No  Don’t know/Can’t remember  No answer | 0 |
| Does your home have nets on the windows and/or doors? | Yes, in all | 1 |
|  | Yes, in some | 0.5 |
|  | No  Don’t know/Can’t remember  No answer | 0 |
| Do you have pet animals? | Yes – other(s)  No | 1,5 |
|  | Yes – dog(s)  Don’t know/Can’t remember  No answer | 0 |
| Do(es) your dog(s) spend time outdoors between dusk and dawn? | No | 0.5 |
|  | Yes – in the garden/backyard  Yes – on the street  Yes – in a forest/bush area  Yes – other  Don’t know/Can’t remember  No answer | 0 |
| Do(es) your dog(s) use insect repellent/insecticide products? | Yes, all year round | 0.5 |
|  | Yes, in some months of the year | 0.25 |
|  | No  Don’t know/Can’t remember  No answer | 0 |
| Is/are your dog(s) regularly seen by a veterinarian? | Yes – more than once per year  Yes – approximately once per year | 0.5 |
|  | Yes – every 2 years | 0.25 |
|  | No  Don’t know/Can’t remember  No answer | 0 |
