# Supplementary figures and images for: Knowledge, perceptions and practices of health students and professionals regarding leishmaniasis in Portugal: a cross-sectional study
Source: Parasit Vectors. 2023 Oct 25;16:381. doi: 10.1186/s13071-023-05982-z (PMC10598964; doi:10.1186/s13071-023-05982-z)

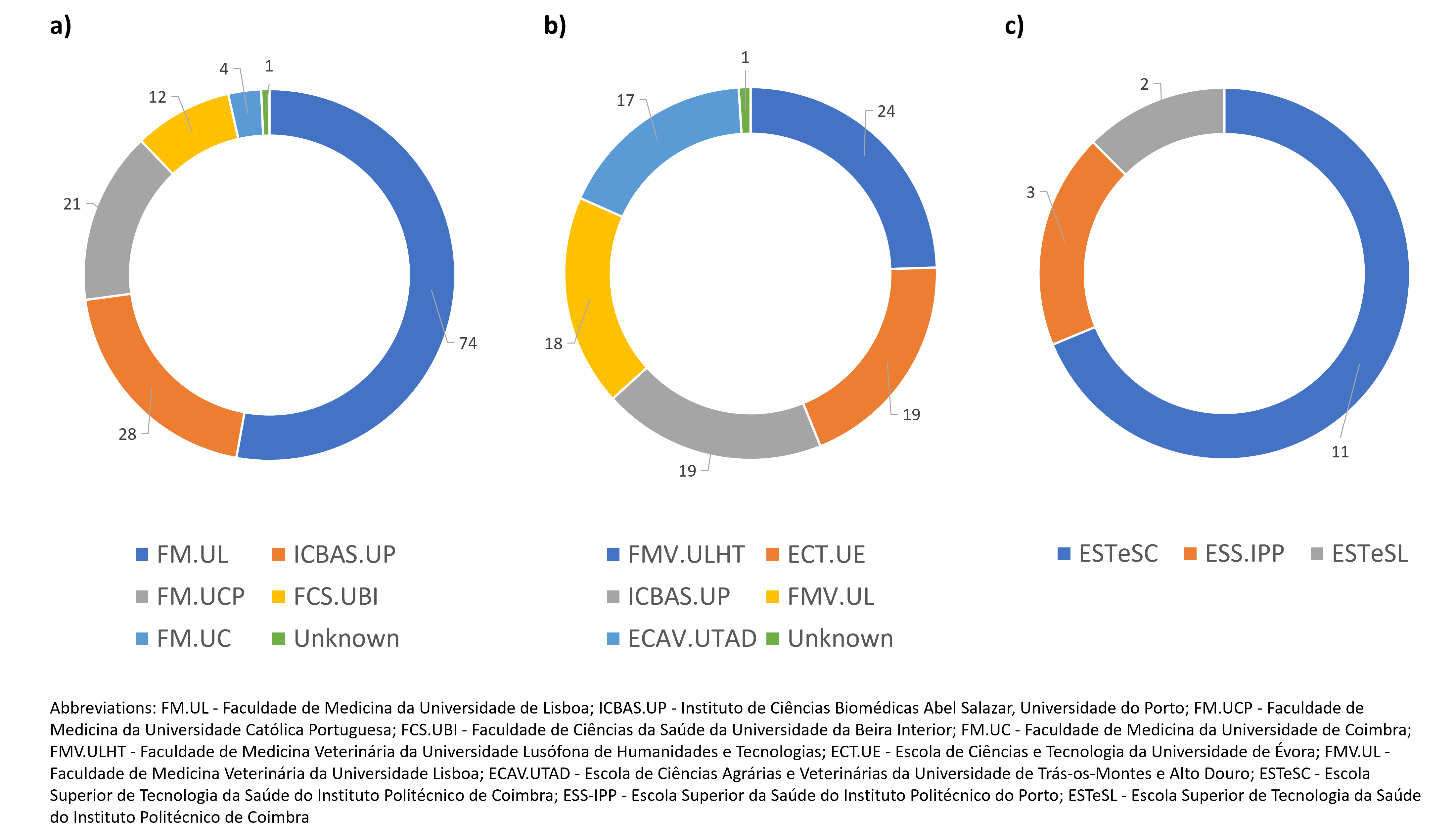

Supplement: Supplementary file 3 — Additional file 3: Figure S2. Distribution of students by university and faculty of study for: a Integrated Master’s in Medicine, b Integrated Master’s in Veterinary Medicine, c Bachelor’s in Environmental Health [file 13071_2023_5982_MOESM3_ESM.png]

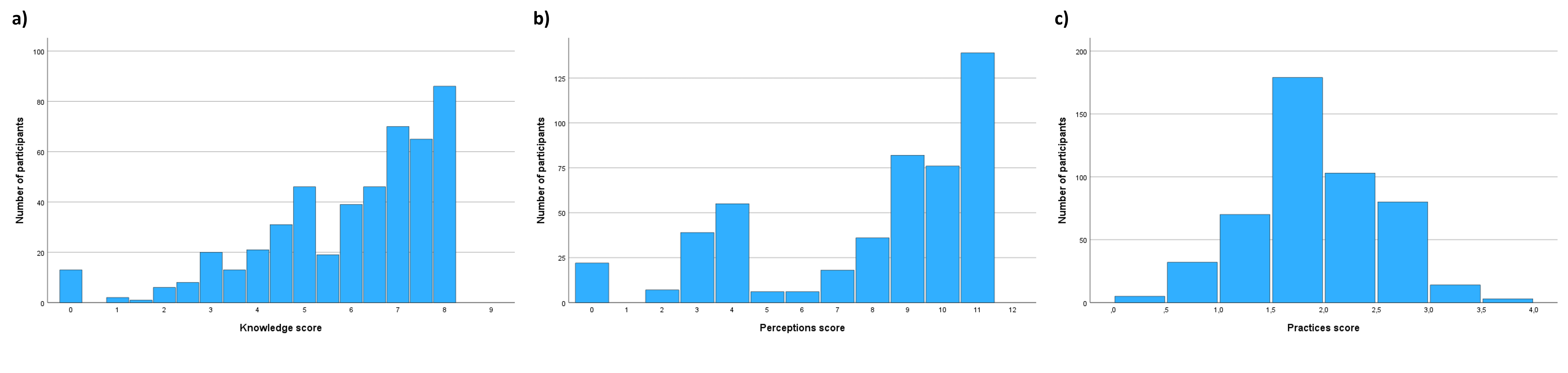

Supplement: Supplementary file 4 — Additional file 4: Figure S3. Distribution of individual: a knowledge scores, b perceptions scores, c practices scores [file 13071_2023_5982_MOESM4_ESM.png]
